# Feedback and Financial Incentives for Reducing Cell Phone Use While Driving: A Randomized Clinical Trial

**DOI:** 10.1001/jamanetworkopen.2024.20218

**Published:** 2024-07-10

**Authors:** M. Kit Delgado, Jeffrey P. Ebert, Ruiying A. Xiong, Flaura K. Winston, Catherine C. McDonald, Roy M. Rosin, Kevin G. Volpp, Ian J. Barnett, Dylan S. Small, Douglas J. Wiebe, Dina Abdel-Rahman, Jessica E. Hemmons, Rafi Finegold, Benjamin Kotrc, Emma Radford, William J. Fisher, Kristen L. Gaba, William C. Everett, Scott D. Halpern

**Affiliations:** 1 Department of Emergency Medicine, Perelman School of Medicine, University of Pennsylvania, Philadelphia; 2 Department of Biostatistics, Epidemiology, and Informatics, University of Pennsylvania, Philadelphia; 3 Penn Medicine Nudge Unit, Philadelphia, Pennsylvania; 4 Center for Health Incentives and Behavioral Economics, University of Pennsylvania, Philadelphia; 5 Penn Injury Science Center, Philadelphia, Pennsylvania; 6 Center for Injury Research and Prevention, Children's Hospital of Philadelphia, Philadelphia; 7 Department of Medicine, Perelman School of Medicine, University of Pennsylvania, Philadelphia; 8 Department of Pediatrics, University of Pennsylvania, Philadelphia; 9 Department of Family Medicine and Community Health, School of Nursing, University of Pennsylvania, Philadelphia; 10 Department of Medical Ethics and Health Policy, University of Pennsylvania, Philadelphia; 11 Department of Statistics and Data Science, The Wharton School, University of Pennsylvania, Philadelphia; 12 Department of Epidemiology, School of Public Health, University of Michigan, Ann Arbor; 13 TrueMotion Inc (acquired by Cambridge Mobile Telematics), Cambridge, Massachusetts; 14 The Progressive Casualty Insurance Company, Mayfield Village, Ohio

## Abstract

**Importance:**

Handheld phone use while driving is a major factor in vehicle crashes. Scalable interventions are needed to encourage drivers not to use their phones.

**Objective:**

To test whether interventions involving social comparison feedback and/or financial incentives can reduce drivers’ handheld phone use.

**Design, Setting, and Participants:**

In a randomized clinical trial, interventions were administered nationwide in the US via a mobile application in the context of a usage-based insurance program (Snapshot Mobile application). Customers were eligible to be invited to participate in the study if enrolled in the usage-based insurance program for 30 to 70 days. The study was conducted from May 13 to June 30, 2019. Analysis was completed December 22, 2023.

**Interventions:**

Participants were randomly assigned to 1 of 6 trial arms for a 7-week intervention period: (1) control; (2) feedback, with weekly push notification about their handheld phone use compared with that of similar others; (3) standard incentive, with a maximum $50 award at the end of the intervention based on how their handheld phone use compared with similar others; (4) standard incentive plus feedback, combining interventions of arms 2 and 3; (5) reframed incentive plus feedback, with a maximum $7.15 award each week, framed as participant’s to lose; and (6) doubled reframed incentive plus feedback, a maximum $14.29 weekly loss-framed award.

**Main Outcome and Measure:**

Proportion of drive time engaged in handheld phone use in seconds per hour (s/h) of driving. Analyses were conducted with the intention-to-treat approach.

**Results:**

Of 17 663 customers invited by email to participate, 2109 opted in and were randomized. A total of 2020 drivers finished the intervention period (68.0% female; median age, 30 [IQR, 25-39] years). Median baseline handheld phone use was 216 (IQR, 72-480) s/h. Relative to control, feedback and standard incentive participants did not reduce their handheld phone use. Standard incentive plus feedback participants reduced their use by −38 (95% CI, −69 to −8) s/h (*P* = .045); reframed incentive plus feedback participants reduced their use by −56 (95% CI, −87 to −26) s/h (*P* < .001); and doubled reframed incentive plus feedback participants reduced their use by −42 s/h (95% CI, −72 to −13 s/h; *P* = .007). The 5 active treatment arms did not differ significantly from each other.

**Conclusions and Relevance:**

In this randomized clinical trial, providing social comparison feedback plus incentives reduced handheld phone use while individuals were driving.

**Trial Registration:**

ClinicalTrials.gov Identifier: NCT03833219

## Key Points

**Question:**

Can behavioral interventions decrease handheld cell phone–based driver distraction?

**Findings:**

In this randomized clinical trial with 2020 participating auto insurance customers, the median baseline level of handheld phone while driving was 216 seconds per hour. Those randomized to interventions combining social comparison feedback and financial incentives reduced their handheld phone use while driving by 15% to 21% relative to the control group.

**Meaning:**

The findings of this study suggest that auto insurers could incorporate these interventions into behavior-based insurance plans and potentially reduce distracted driving at scale and therefore crash risk in the population.

## Teaser

This randomized clinical trial evaluates whether an app that monitors handheld phone use while driving and provides feedback and/or financial incentives to drivers reduces rates of distracted driving.

## Introduction

In 2021 in the US, there were 804 928 motor vehicle crashes caused by distracted driving, resulting in 3522 deaths and 362 415 injures.^1^ Handheld manipulation of cell phones while driving is a leading cause of distracted driving crashes; when drivers look away from the road for more than 2 seconds, their crash risk increases exponentially.^2,3,4^ Increasing crash claims from distracted driving contributed to a 30% increase in auto insurance premiums between 2011 and 2020.^5^ In response, most of the top 10 US auto insurers now offer usage-based insurance (UBI) in which a smartphone app or a plug-in telematics device monitors a person’s driving behaviors to assess risk and price their insurance policy accordingly.^6^ Companies that offer UBI have been able to decrease their insurance losses by better predicting future claims and calibrating their pricing.^7^ The newest generation of smartphone telematics apps has the ability to capture cell phone use of the driver, applying classification algorithms to distinguish between driver and passenger use.^8^ The global UBI market is projected to grow from $58 billion in 2023 to $174 billion by 2030.^9^

This transition to pricing auto insurance based on observed driving behavior presents an opportunity to encourage safer driving and reduce crashes at scale. The status quo for UBI programs is pay-how-you-drive in which driving behaviors are monitored (eg, hard braking, late-night driving, speeding, and now cell phone use) and customers receive a personalized rate on the next 6-month insurance policy with potential for cash back rewards every 6 months. Among UBI programs, the fastest growing market segment is manage-how-you-drive, in which apps deliver feedback aimed at curbing risky behaviors.^9^ By applying insights from behavioral science, UBI could be redesigned to be more effective at reducing risky behaviors, such as distracted driving owing to cell phone use.^10,11,12^ We conducted a randomized clinical trial to evaluate the effectiveness of 5 behavioral interventions for reducing drivers’ handheld cell phone use.

## Methods

### Study Setting and Dates

The trial protocol was approved by the institutional review board of the University of Pennsylvania and is included with the preregistered statistical analysis plan in Supplement 1. The recruitment of study participants was conducted by the Progressive Causality Insurance Company, and the randomization and delivery of interventions and the collection of data were conducted by Progressive’s mobile technology vendor, TrueMotion (acquired by Cambridge Mobile Telematics after study period), via the Snapshot Mobile Application. Deidentified data on individuals who agreed to participate were transmitted to the University of Pennsylvania for analysis. The intervention took place between May 13 and June 30, 2019; participants continued to be monitored until the end of their Snapshot rating period. Deidentified data were transmitted to the University of Pennsylvania for analysis. We followed the Consolidated Standards of Reporting Trials (CONSORT) reporting guideline for randomized trials.

### Participants

The insurance customers were eligible if they had been enrolled in the UBI program for between 30 and 70 days and resided in 1 of 42 US states or the District of Columbia. Customers were ineligible to be recruited if they lived in a state in which cell phone use was factored into the insurance rating, if the app was not updated to enable push notification, or if the app was not collecting trip data. Eligible customers received up to 2 emails from the company inviting them to participate. The insurance company only solicited single-vehicle/single-driver policyholders, and each email included a unique code that was required to register for the study. Interested customers clicked a link, which took them to a webpage describing the study conducted in collaboration with the University of Pennsylvania. Customers were informed that the purpose of the study was to test whether the app can help reduce distracted driving. Participants were guaranteed $20 for survey completion, with a chance to earn an additional $50 to $100 depending on the group to which they were randomly assigned. It was emphasized that money earned through the study was distinct from any app discount they might get. At the time of the study, handheld phone use was measured by the app but not factored in their insurance rating. Customers were informed that they might receive app notifications while participating. Those who agreed to participate provided their email address and continued to an online intake survey.

### Interventions

The company randomly assigned participants in an equal allocation ratio to 1 of 6 arms using a random number generator for a 7-week intervention period. The first arm served as the control group. Control participants’ phone use was measured by the app, but they were given no feedback about how they compared with others and no incentive to reduce use.

In the second (feedback) arm, participants received weekly push notifications about how their handheld phone use compared with that of similar participants. Given that more than 80% of people believe they are better-than-average drivers^13^ and phone use while driving is often an unconscious habit,^14^ we theorized this feedback would raise participants’ awareness of their own distracted driving behavior and motivate them to reduce it. If weekly handheld phone use was greater than the historical median (worse than average) for app customers with similar demographic characteristics (age, sex, marital status, and urban, suburban, or rural residence), their notification said that most customers used their phone less than they did. If weekly use was less than or equal to the median but greater than the 10th percentile (better than average), their notification said that if they used their phone less, they would be one of the company’s best drivers. If their use was less than or equal to the 10th percentile (top performer), their notification said they were one of the best drivers and encouraged them to keep up the good work. Participants also received an encouraging notification the first time they had a driving trip without handheld phone use each week.

The third (standard incentive) arm was designed to be like a typical pay-how-you-drive UBI program, with an incentive (maximum $50) awarded at the end of the intervention period and no weekly social comparison feedback. Participants were told that if they finished as a top performer, they would receive the full $50; if better than average, they would receive $25; and if worse than average, they would get no money.

In the fourth (standard incentive plus feedback) arm, participants received both the standard incentive (maximum $50) and the weekly social comparison feedback and encouragement for their first drive without handheld phone use. They also got weekly notifications that they needed to keep their phone use low to earn an incentive payment at the end of the intervention period.

The fifth (reframed incentive plus feedback) arm was designed with insights from behavioral economics. First, we turned present bias—a preference for immediate rewards that is stronger among people who engage in phone use while driving—to our advantage.^15^ Instead of offering a lump sum at the end of the intervention period, we offered smaller weekly incentives totaling the same amount. Participants were eligible to receive the full weekly incentive ($7.15) if they finished as a top performer for the week, and half ($3.58) if they finished outside the top but better than average. Second, we capitalized on loss aversion, the avoidance of losses over equivalent gains, by telling participants to expect a weekly payment while warning that it would be withheld if they did not keep their phone use low.^16^ Third, we leveraged regret aversion, the preference for minimizing regret in decision-making, by presenting a running table of money lost each week for not achieving top performer status.^17^ Fourth, we made use of the fresh start effect, in which people are more likely to take action toward a goal after a temporal landmark, by telling participants who did not finish as a top performer that they got a fresh start to earn money the next week.^18^ All participants in this arm also received the weekly social comparison feedback and encouragement for their first drive without handheld phone use.

The sixth (doubled reframed incentive plus feedback) arm was designed to test the dose-response effects of larger incentives. The intervention was identical to the reframed incentive plus feedback arm, except the total incentive was doubled (maximum $100, with weekly incentives of $14.29 for top performers and $7.15 for better than average). To summarize:

1. Control: no incentive; no feedback.
2. Feedback: no incentive; social comparison feedback.
3. Standard incentive: up to $50 delayed, gain-framed; no feedback.
4. Standard incentive plus feedback: up to $50 delayed, gain-framed; social comparison feedback.
5. Reframed incentive plus feedback: up to $7.15/wk, loss-framed; social comparison feedback.
6. Doubled reframed incentive plus feedback: up to $14.29/wk, loss-framed; social comparison feedback.

During the trial we also tested a nudge designed to encourage situational self-control by removing the temptation to respond to text messages and app notifications.^19^ Participants with iPhones in all arms, including the control group, received 3 push notifications inviting them to set their phone’s do not disturb while driving feature to activate automatically whenever driving was detected. At the time of this trial, a comparable setting was not widely available on Android phones; participants with Android phones thus served as a naturally occurring, albeit nonrandom, control group.

After the intervention period, participants’ phone use and driving behavior continued to be monitored for the remainder of their 150-day rating period, allowing us to test whether any behavior change persisted after incentives and feedback ceased. Because customers were eligible to participate if enrolled for between 30 and 70 days, the postintervention follow-up period remaining in their rating period varied in length with a mean (SD) of 40.0 (12.1) days. All study investigators were blinded to arm assignments until after the intervention period.

### Outcomes and Measures

Primary and secondary outcomes were measured by the Snapshot Mobile app. The primary outcome was proportion of drive time engaged in handheld phone use during the intervention period measured in s/h of driving. Secondary outcomes, for which we made no a priori hypotheses, were (1) proportion of drive time engaged in the subcomponents of handheld phone use (call vs noncall, eg, texting, swiping, or typing), (2) proportion of drive time engaged in handsfree phone use, (3) hard braking events per 100 miles of driving, and (4) hard acceleration events per 100 miles of driving.

Prespecified covariates were obtained from the insurance company and the intake survey. The company provided each participant’s age, sex, marital status, and urban, suburban, or rural residence, as well as information needed to determine whether they resided in a state with a universal handheld phone ban, their length of enrollment in the app at baseline, their proportion of baseline drive time engaged in handheld phone use, their baseline mean hours of driving per week, and their baseline proportion of handheld phone use due to calls. To characterize the representativeness of the study population, the intake survey asked about additional demographic variables (race and ethnicity, household income, and educational level; to create mutually exclusive race categories for analytic purposes, participants who indicated Black as an identity were categorized as Black, those who solely indicated White as an identity were categorized as White, and all other participants were categorized as other, including Asian, American Indian or Alaska Native, multiracial, and Native Hawaiian or Other Pacific Islander) and factors theorized to be associated with handheld phone use while driving (phone type, use of automated do not disturb while driving setting, Bluetooth/USB car connectivity, presence of dashboard touchscreen, frequency of letting a passenger use the phone, frequency of riding as a passenger, traffic violations in the previous 5 years, and car crashes in the previous 5 years).

### Statistical Analysis

For the primary analysis, we used R, version 4.3.2 (R Foundation for Statistical Computing) to conduct fractional regression with a logit link.^20^ The model was adjusted with the prespecified covariates. The regression coefficients and effect sizes in fractional logistic regression cannot be intuitively interpreted as is. We therefore calculated average marginal effects representing the change in the proportion of driving time observed in each of the active treatment arms compared with the control arm, using the delta method to calculate 95% CIs. We then converted this to s/h of driving time for a more intuitive, policy-relevant interpretation of the effect sizes.

Due to a survey administration error, information was unavailable about race (17.9%), ethnicity (17.0%), income (23.8%), and educational level (10.3%) for a substantial number of participants; these participants were included in analyses with missing covariates dummy-coded with a missing value of 1. Missingness for other covariates was low (0.0%-5.0%); participants missing data for these other covariates were excluded from the complete case analysis reported herein.

Each of the 5 treatment arms was compared with the control group. We determined a priori 5 other between-group comparisons for a total of 10 contrasts. We estimated that to have a power of 0.80 to detect a relative reduction of 25% from the historical baseline mean of 288 s/h of driving (a reduction of 72 s/h of driving) we would need a sample of at least 1842 participants (301 per arm). This calculation was based on our prespecified plan to use the Holm method to handle multiple comparisons by sequentially testing the significance of each contrast against progressively less restrictive α levels, maintaining a family-wise type I error rate of .05 (2-sided). Analyses were conducted with the intention-to-treat approach.

We also conducted 3 prespecified exploratory effect modification analyses. First, we examined whether interventions were more effective for participants in the highest quartile of baseline handheld phone use. We hypothesized that their reduction in handheld phone use would be greater because they had the greatest opportunity for improvement. Second, we examined whether interventions were more effective for participants with iPhones. We hypothesized that iPhone users would experience greater reductions in handheld phone use because they received notifications encouraging them to activate the do not disturb while driving feature. Third, we examined whether intervention effectiveness varied by participants’ baseline proportion of handheld phone use due to phone calls. We hypothesized that those above the median for proportion of call use would have greater reductions in handheld phone use due to widely available technologies for taking calls handsfree.

The app has an algorithm that classifies each trip as either driver or passenger for the purposes of insurance rating. Customers can correct a classification in the app (eg, change a driver trip to passenger) up to 5 days after the trip. Only trips that were classified as a driver trip and not corrected or classified as a passenger trip and corrected were included in the primary analysis. Because treatment arm participants stood to gain by correcting driver trips during which they used their phone, we performed a prespecified sensitivity analysis that included all and only trips originally classified by the algorithm as driver.

To determine whether any changes in behavior were sustained after interventions ended, we compared handheld phone use by randomization arm in the postintervention period using the same statistical model as our primary analysis. Analysis was completed December 22, 2023. After Holm adjustment for multiple comparisons, *P* < .05 was considered significant.

## Results

Of 17 663 customers invited by email to participate, 2109 opted in and were randomized. A total of 2020 individuals from 35 US states participated and finished the intervention period (eFigure 1 in Supplement 2 provides the CONSORT diagram and eTable 1 in Supplement 2 reports a comparison with individuals who did not enroll). Median participant age was 30 (IQR, 25-39) years (Table 1). Overall, 1373 participants were female (68.0%) and 647 were male (32.0%). Of the total population, 20.1% of the participants were Black, 9.5% were Hispanic, and 55.0% were White.

**Table 1.** Participant Characteristics

Characteristic	Participants by trial arm, No. (%)						
	Overall (N = 2020)	Control (n = 333)	Feedback (n = 340)	Standard incentive (n = 331)	Standard incentive plus feedback (n = 336)	Reframed incentive plus feedback (n = 339)	Double reframed incentive plus feedback (n = 341)
Age, median (IQR), y	30 (25-39)	30 (25-39)	30 (25-37)	31 (25-40)	30 (25-38)	31 (25-42)	30 (26-38)
Sex							
Female	1373 (68.0)	242 (72.7)	230 (67.6)	224 (67.7)	223 (66.4)	223 (65.8)	231 (67.7)
Male sex	647 (32.0)	91 (27.3)	110 (32.4)	107 (32.3)	113 (33.6)	116 (34.2)	110 (32.3)
Single marital status	1745 (86.4)	290 (87.1)	299 (87.9)	283 (85.5)	286 (85.1)	288 (85.0)	299 (87.7)
Race and ethnicity^a^							
Black	407 (20.1)	68 (20.4)	66 (19.4)	74 (22.4)	62 (18.5)	74 (21.8)	63 (18.5)
White	1112 (55.0)	184 (55.3)	173 (50.9)	175 (52.9)	189 (56.2)	200 (59.0)	191 (56.0)
Other	139 (6.9)	20 (6.0)	32 (9.4)	22 (6.6)	21 (6.2)	21 (6.2)	23 (6.7)
Missing	362 (17.9)	61 (18.3)	69 (20.3)	60 (18.1)	64 (19.0)	44 (13.0)	64 (18.8)
Hispanic							
No	1486 (73.6)	243 (73.0)	242 (71.2)	245 (74.0)	247 (73.5)	266 (78.5)	243 (71.3)
Yes	191 (9.5)	34 (10.2)	32 (9.4)	30 (9.1)	27 (8.0)	30 (8.8)	38 (11.1)
Missing	343 (17.0)	56 (16.8)	66 (19.4)	56 (16.9)	62 (18.5)	43 (12.7)	60 (17.6)
Annual household income, $							
<50 000	950 (47.0)	165 (49.5)	149 (43.8)	150 (45.3)	150 (44.6)	173 (51.0)	163 (47.8)
50 000-100 000	462 (22.9)	71 (21.3)	74 (21.8)	86 (26.0)	82 (24.4)	75 (22.1)	74 (21.7)
>100 000	128 (6.3)	21 (6.3)	25 (7.4)	15 (4.5)	26 (7.7)	24 (7.1)	17 (5.0)
Missing	480 (23.8)	76 (22.8)	92 (27.1)	80 (24.2)	78 (23.2)	67 (19.8)	87 (25.5)
Educational level							
High school or less	430 (21.3)	70 (21.0)	77 (22.6)	59 (17.8)	78 (23.2)	66 (19.5)	80 (23.5)
Some college	626 (31.0)	96 (28.8)	114 (33.5)	100 (30.2)	98 (29.2)	108 (31.9)	110 (32.3)
College degree and above	756 (37.4)	135 (40.5)	110 (32.4)	128 (38.7)	128 (38.1)	132 (38.9)	123 (36.1)
Missing	208 (10.3)	32 (9.6)	39 (11.5)	44 (13.3)	32 (9.5)	33 (9.7)	28 (8.2)
Residence							
Rural	404 (20.0)	65 (19.5)	62 (18.2)	60 (18.1)	79 (23.5)	66 (19.5)	72 (21.1)
Suburban	1243 (61.5)	208 (62.5)	202 (59.4)	209 (63.1)	206 (61.3)	215 (63.4)	203 (59.5)
Urban	373 (18.5)	60 (18.0)	76 (22.4)	62 (18.7)	51 (15.2)	58 (17.1)	66 (19.4)
State has universal handheld ban	630 (31.2)	99 (29.7)	107 (31.5)	107 (32.3)	104 (31.0)	113 (33.3)	100 (29.3)
Handheld phone use, min/h driving at baseline, median (IQR)	3.6 (1.2-8.0)	4.2 (1.4-8.3)	3.4 (1.2-8.1)	3.6 (1.2-8.6)	3.9 (1.4-8.2)	3.2 (1.1-7.1)	3.4 (1.1-7.5)
Drive time at baseline, h/wk, median (IQR)	6.7 (3.9-10.4)	7.1 (4.3-10.6)	6.6 (3.9-9.9)	7.0 (3.9-10.7)	6.7 (4.0-10.9)	6.2 (3.8-9.9)	6.6 (3.9-9.8)
Proportion of handheld phone use due to calls at baseline, median (IQR)	0.095 (0.000-1.000)	0.102 (0.000-0.968)	0.094 (0.000-1.000)	0.102 (0.000-1.000)	0.098 (0.000-0.879)	0.084 (0.000-0.954)	0.083 (0.000-1.000)
Length of enrollment during baseline, median (IQR)	50.0 (39.0-60.0)	50.0 (39.0-60.0)	50.0 (40.0-59.0)	49.0 (38.0-59.0)	50.0 (39.8-59.0)	51.0 (39.0-60.0)	50.0 (41.0-61.0)
iPhone	1327 (65.7)	225 (67.6)	224 (65.9)	233 (70.4)	214 (63.7)	213 (62.8)	218 (63.9)
Baseline use of automated do not disturb							
No	1389 (68.8)	226 (67.9)	230 (67.6)	229 (69.2)	238 (70.8)	234 (69.0)	232 (68.0)
Yes	592 (29.3)	97 (29.1)	107 (31.5)	99 (29.9)	88 (26.2)	97 (28.6)	104 (30.5)
Missing	39 (1.9)	10 (3.0)	3 (0.9)	3 (0.9)	10 (3.0)	8 (2.4)	5 (1.5)
Bluetooth/USB car connectivity							
No	358 (17.7)	54 (16.2)	62 (18.2)	63 (19.0)	57 (17.0)	53 (15.6)	69 (20.2)
Yes	1627 (80.5)	270 (81.1)	276 (81.2)	264 (79.8)	269 (80.1)	278 (82.0)	270 (79.2)
Missing	35 (1.7)	9 (2.7)	2 (0.6)	4 (1.2)	10 (3.0)	8 (2.4)	2 (0.6)
Dashboard touchscreen in car							
No	1291 (63.9)	214 (64.3)	225 (66.2)	215 (65.0)	201 (59.8)	218 (64.3)	218 (63.9)
Yes	695 (34.4)	110 (33.0)	112 (32.9)	113 (34.1)	125 (37.2)	116 (34.2)	119 (34.9)
Missing	34 (1.7)	9 (2.7)	3 (0.9)	3 (0.9)	10 (3.0)	5 (1.5)	4 (1.2)
Frequency letting passenger use phone							
Never	673 (33.3)	107 (32.1)	98 (28.8)	125 (37.8)	112 (33.3)	117 (34.5)	114 (33.4)
1-2 d	657 (32.5)	106 (31.8)	117 (34.4)	113 (34.1)	99 (29.5)	108 (31.9)	114 (33.4)
≥3 d	653 (32.3)	110 (33.0)	123 (36.2)	90 (27.2)	114 (33.9)	108 (31.9)	108 (31.7)
Missing	37 (1.8)	10 (3.0)	2 (0.6)	3 (0.9)	11 (3.3)	6 (1.8)	5 (1.5)
Frequency riding as passenger							
Never	481 (23.8)	76 (22.8)	75 (22.1)	90 (27.2)	77 (22.9)	77 (22.7)	86 (25.2)
1-2 d	798 (39.5)	129 (38.7)	135 (39.7)	127 (38.4)	145 (43.2)	133 (39.2)	129 (37.8)
≥3 d	704 (34.9)	118 (35.4)	128 (37.6)	111 (33.5)	104 (31.0)	123 (36.3)	120 (35.2)
Missing	37 (1.8)	10 (3.0)	2 (0.6)	3 (0.9)	10 (3.0)	6 (1.8)	6 (1.8)
Traffic violations in prior 5 y							
0	1169 (57.9)	203 (61.0)	191 (56.2)	205 (61.9)	179 (53.3)	200 (59.0)	191 (56.0)
1	475 (23.5)	68 (20.4)	83 (24.4)	69 (20.8)	81 (24.1)	81 (23.9)	93 (27.3)
2	218 (10.8)	29 (8.7)	36 (10.6)	39 (11.8)	42 (12.5)	35 (10.3)	37 (10.9)
≥3	57 (2.8)	14 (4.2)	14 (4.1)	9 (2.7)	8 (2.4)	4 (1.2)	8 (2.3)
Missing	101 (5.0)	19 (5.7)	16 (4.7)	9 (2.7)	26 (7.7)	19 (5.6)	12 (3.5)
Car crashes in prior 5 y							
0	1168 (57.8)	196 (58.9)	206 (60.6)	184 (55.6)	194 (57.7)	186 (54.9)	202 (59.2)
1	540 (26.7)	85 (25.5)	85 (25.0)	99 (29.9)	84 (25.0)	99 (29.2)	88 (25.8)
2	185 (9.2)	26 (7.8)	30 (8.8)	35 (10.6)	31 (9.2)	30 (8.8)	33 (9.7)
≥3	29 (1.4)	7 (2.1)	3 (0.9)	4 (1.2)	2 (0.6)	7 (2.1)	6 (1.8)
NA	98 (4.9)	19 (5.7)	16 (4.7)	9 (2.7)	25 (7.4)	17 (5.0)	12 (3.5)

Abbreviation: NA, not applicable.

^a^ To create mutually exclusive race categories for analytic purposes, participants who indicated Black as an identity were categorized as Black, those who solely indicated White as an identity were categorized as White, and all other participants were categorized as other, which included Asian, American Indian or Alaska Native, multiracial, and Native Hawaiian or Other Pacific Islander.

**Figure 1.**
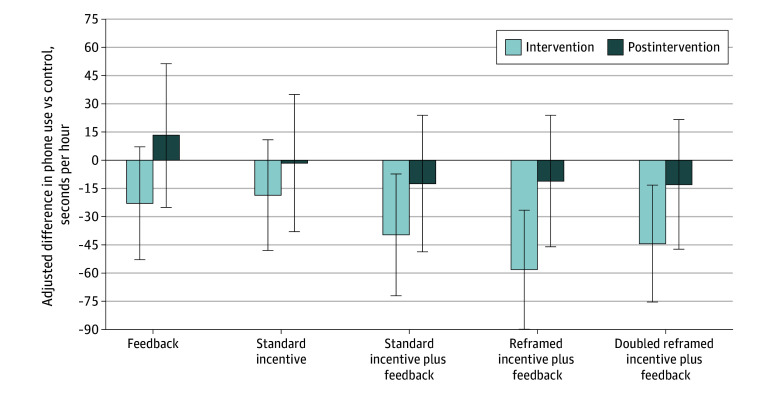
Primary Outcome: Differences in Mean Handheld Phone Use Compared With Control Error bars indicate 95% CIs. Marginal effects were estimated using a prespecified fractional logistic regression model adjusted for 19 prespecified covariates listed in Table 1. Details on the model are available in the statistical analysis plan included in Supplement 1. Unadjusted outcomes by arm are presented in eFigure 2 in Supplement 2.

Median baseline handheld phone use was 216 (IQR, 72-480) s/h. After correcting for the number of planned comparisons, neither the feedback intervention nor the standard incentive (ie, delayed) intervention produced significant reductions in handheld phone use compared with the control group (Table 2). However, combining both interventions, as the standard incentive plus feedback arm did, reduced handheld phone use by −38 (95% CI, −69 to −8) s/h of driving (*P* = .045) (Figure 1). This was a 14.5% reduction compared with the control group. In the reframed incentive plus feedback arm, which offered the same incentive amount but delivered it according to behavioral economics principles, participants reduced their handheld phone use by −56 (95% CI, −87 to −26) s/h of driving (*P* < .001), a relative reduction of 21.2%. Participants in the doubled reframed incentive plus feedback arm, which was identical except for its larger weekly incentives, reduced their handheld phone use by −42 (95% CI, −72 to −13) s/h (*P* = .007), a relative reduction of 16.0%. There were no statistically significant differences among prespecified comparisons of active interventions against each other.

**Table 2.** Holm Sequential Analysis of 10 Prespecified Trial Group Comparisons^a^

Comparison	Raw *P* value	Rank	Remaining contrasts	Holm threshold	Adjusted *P* value
Feedback vs control	.09	4	7	.007	.64
Standard incentive vs control	.16	6	5	.01	.82
Standard incentive plus feedback vs control	.006	3	8	.006	.045
Reframed incentive plus feedback vs control	<.001	1	10	.005	<.001
Double reframed incentive plus feedback vs control	<.001	2	9	.006	.007
Standard incentive vs feedback	.75	10	1	.050	.75
Standard incentive plus feedback vs standard incentive	.14	5	6	.009	.85
Standard incentive plus feedback vs feedback	.25	8	3	.017	.74
Reframed incentive plus feedback vs standard incentive plus feedback	.17	7	4	.013	.67
Double reframed incentive plus feedback vs reframed incentive plus feedback	.24	9	2	.025	.49

^a^ Adjusted *P* values may be directly compared with a 2-sided α threshold of .05. Differences between treatment arms were estimated using a prespecified fractional logistic regression model adjusted for the prespecified covariates in Table 1. Details on the model are available in the statistical analysis plan included in Supplement 1. Unadjusted outcomes by arm are presented in eTable 2 in Supplement 2.

Unadjusted results for the full sample were similar, as reported in eFigure 2 and eTable 2 in Supplement 2. The sensitivity analysis excluding participant-corrected trips found the same 3 significant results (all *P* < .05). In the postintervention period, none of the reductions in handheld phone use compared with controls remained significant.

**Figure 2.**
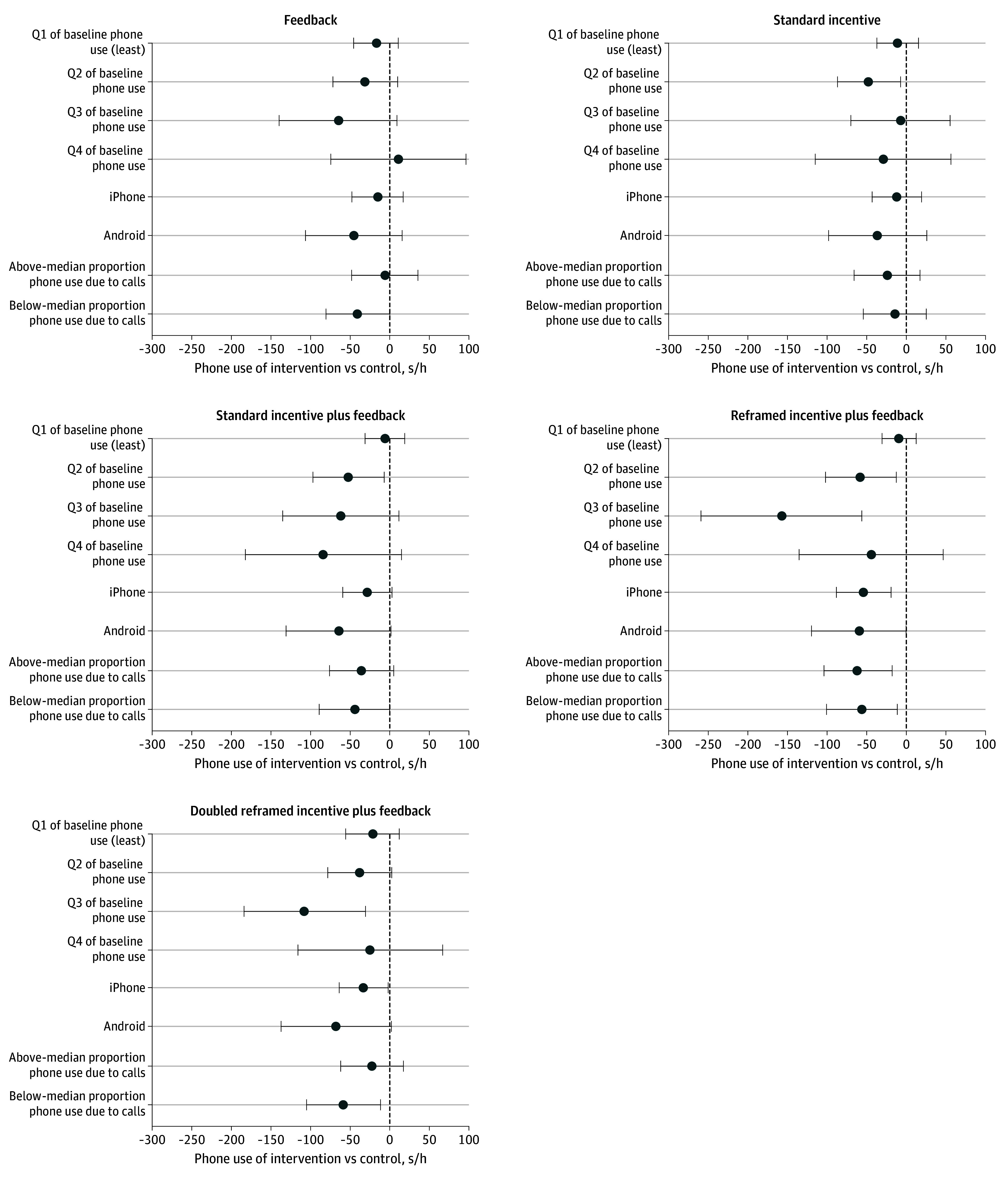
Treatment Effects by Subgroup Mean adjusted differences in handheld phone use compared with controls for the intervention period. Lower values indicate better results. Error bars indicate 95% CIs. Differences between treatment arms were estimated using a prespecified fractional logistic regression model adjusted for the prespecified covariates listed in Table 1. Details on the model are available in the statistical analysis plan included in Supplement 1. Post-intervention treatment effects by subgroup are presented in eFigure 3 in Supplement 2. Q indicates quartile; s/h, seconds per hour.

We explored potential moderators of these effects in 3 preplanned analyses (Figure 2). In the first, we did not find that those with the highest baseline handheld phone use (quartile 4) reduced their use in response to interventions. However, among those with above median baseline use (quartile 3), reframed incentive plus feedback participants reduced their handheld phone use by −157 (95% CI, −258 to −56) s/h compared with controls, and doubled reframed incentive plus feedback participants reduced their use by −108 (95% CI, −184 to −31) s/h. In the second moderator analysis, we found no evidence that iPhone users changed their behavior more than Android users. In the third, we found no evidence that participants whose baseline handheld phone use involved an above-median proportion of call time changed their behavior more.

In analysis of secondary outcomes, we found that the same 3 interventions that reduced handheld phone use overall reduced noncall use; none reduced handheld phone call use (Table 3). None of the interventions reduced handsfree phone use, hard-braking events, or hard-acceleration events (eFigure 3 in Supplement 2).

**Table 3.** Secondary Outcomes

Outcome	Effect size (95% CI)							
	Feedback	Standard incentive		Standard incentive plus feedback		Reframed incentive plus feedback		Doubled reframed incentive plus feedback
**Intervention period**								
All phone use, s/h of driving	−49 (−112 to 15)	−28 (−87 to 31)	−80 (−145 to −16)		−57 (−121 to 7)		−68 (−131 to −6)	
Handheld phone use^a^	−22 (−51 to 6)	−18 (−46 to 10)	−38 (−69 to −8)		−56 (−87 to −26)		−42 (−72 to −13)	
Noncall	−23 (−48 to 2)	−17 (−41 to 7)	−37 (−65 to −9)		−50 (−76 to −23)		−40 (−66 to −14)	
Call	−4 (−13 to 5)	−1 (−9 to 8)	0 (−9 to 8)		−7 (−16 to 2)		−4 (−13 to 5)	
Handsfree use	−14 (−58 to 30)	1 (−41 to 43)	−27 (−71 to 18)		8 (−38 to 54)		−20 (−62 to 23)	
Hard-brake events, per 100 miles of driving	−0.05 (−0.45 to 0.35)	0.01 (−0.39 to 0.42)	−0.08 (−0.49 to 0.32)		0.1 (−0.3 to 0.5)		−0.2 (−0.6 to 0.2)	
Hard-acceleration events, per 100 miles of driving	0.01 (−0.01 to 0.03)	0 (−0.01 to 0.02)	0 (−0.01 to 0.02)		0 (−0.01 to 0.02)		0 (−0.02 to 0.01)	
**Postintervention period**								
All phone use, s/h of driving	21 (−57 to 98)	34 (−45 to 112)	−11 (−90 to 68)		34 (−46 to 114)		−12 (−83 to 59)	
Handheld phone use	14 (−23 to 51)	−1 (−36 to 34)	−11 (−46 to 23)		−10 (−44 to 23)		−12 (−45 to 22)	
Noncall	−3 (−35 to 29)	4 (−27 to 35)	−21 (−53 to 10)		−13 (−43 to 18)		−19 (−49 to 11)	
Call	10 (−3 to 24)	−4 (−13 to 4)	8 (−3 to 19)		1 (−9 to 10)		5 (−6 to 16)	
Handsfree use	5 (−53 to 63)	36 (−22 to 93)	7 (−51 to 64)		39 (−21 to 99)		−4 (−56 to 48)	
Hard-brake events, per 100 miles of driving	−0.13 (−0.65 to 0.39)	−0.13 (−0.65 to 0.38)	0.06 (−0.46 to 0.57)		−0.38 (−0.89 to 0.14)		0.11 (−0.41 to 0.62)	
Hard-acceleration events, per 100 miles of driving	0 (−0.03 to 0.03)	−0.01 (−0.04 to 0.02)	0 (−0.03 to 0.03)		−0.01 (−0.04 to 0.02)		−0.01 (−0.05 to 0.02)	

^a^ Primary outcome. Differences between treatment arms were estimated using a prespecified fractional logistic regression model for phone-related outcomes adjusted for 19 prespecified covariates listed in Table 1. Details on the model are available in the statistical analysis plan included in Supplement 1. Hard braking and hard acceleration event difference were estimated with a linear regression adjusted for the prespecified covariates in Table 1.

## Discussion

To our knowledge, this was among the first large-scale randomized clinical trials to investigate the separate and joint effects of incentives and behavioral science interventions designed to reduce handheld phone use while driving. It was conducted pragmatically (ie, within one of the largest auto insurer customer bases with a diverse, national sample), demonstrating that these types of interventions could be scaled to millions of drivers. Advances in smartphone telematics made it possible to examine changes in observed risky driving behavior instead of relying on self-reports. Results showed that providing weekly social comparison feedback in conjunction with a UBI-like delayed incentive led to a 15% decrease in drivers’ handheld phone use. Pairing the social comparison feedback with more frequent, loss-framed incentives of the same total value led to a nonsignificantly greater 21% decrease. Neither feedback alone nor a delayed incentive alone changed drivers’ behavior.

The average per-participant cost of incentives for the most successful treatment was $26, less than standard auto-insurance discounts for safe driving records. Doubling the incentive amount did not yield greater behavior change. While monetary incentives play an important role in helping people to not use their phones, their effectiveness may depend less on the amount of money and more on how it is delivered. Our results indicate that a driver’s baseline handheld phone use also matters. Those in the third quartile of baseline use showed the biggest absolute changes in behavior, whereas those in the highest quartile of baseline use did not change. It may be that becoming better than average at avoiding handheld phone use was too challenging a goal for the heaviest users, or that a qualitatively different approach is needed to help them change. Because changing the behavior of the heaviest users would improve road safety the most, future work in this area is critical. In addition, we found that changes in behavior were not sustained after the interventions ended. Further research is needed to determine whether drivers require continual feedback and incentives to sustain behavior change or if augmenting and lengthening the interventions can help drivers build self-sustaining habits.

### Limitations

This study had limitations. First, participants enrolled on an opt-in basis. The resulting sample may have been more receptive to our interventions and willing to change their phone use behavior than the typical UBI customer. Second, we were unable to directly measure how much drivers took their eyes off the road and instead relied on the app’s measurement of handheld phone use while driving as a proxy for driver distraction. Third, the research team did not have access to participant crash claim data to determine whether reductions in handheld phone use translated to reductions in crash risk. Fourth, the results of the prespecified subgroup analyses and secondary outcomes analyses should be interpreted cautiously as these analyses were exploratory and not powered adequately. Furthermore, randomization was not performed at the subgroup level.

## Conclusions

Prior research has found that even a small reduction in a driver’s handheld phone use can have big safety benefits. Taking one’s eyes off the road for 5 seconds—an amount typical of sending or reading a text—increases one’s risk of crash by a factor of nearly 9.^3^ In the US, drivers spend 70 billion hours driving during a typical year.^21^ If delivered at full scale in a sustained fashion, our intervention that reduced hourly handheld phone use by 56 seconds could mean up to 1 billion fewer hours of distracted driving in the US alone. Smartphones have contributed to widespread distracted driving, but the present results suggest that smartphone-enabled UBI programs that reward drivers who do not use their phones while driving hold promise for reducing motor vehicle crashes.

## Notes

Supplement 1.
Trial Protocol and Statistical Analysis Plan

Supplement 2. **eFigure 1.** CONSORT Flow Diagram **eTable 1.** Comparison of Eligible Customers Who Did and Did Not Enroll **eFigure 2.** Differences in Handheld Phone Use Relative to Control (Raw, Unadjusted Outcomes) **eTable 2.** Holm Sequential Analysis of 10 Pre-Specified Trial Group Comparisons (Raw, Unadjusted Outcomes) **eFigure 3.** Treatment Effects by Subgroup (Post-Intervention Period)

Supplement 3.
Data Sharing Statement

## References

[1] National Center for Statistics and Analysis. Distracted driving in 2021 (research note report No. DOT HS 813 443). National Highway Traffic Safety Administration; May 2023.

[2] Klauer SG, Guo F, Simons-Morton BG, Ouimet MC, Lee SE, Dingus TA. Distracted driving and risk of road crashes among novice and experienced drivers. N Engl J Med. 2014;370(1):54-59. doi:10.1056/NEJMsa1204142 24382065 PMC4183154

[3] Simons-Morton BG, Guo F, Klauer SG, Ehsani JP, Pradhan AK. Keep your eyes on the road: young driver crash risk increases according to duration of distraction. J Adolesc Health. 2014;54(5 suppl):S61-S67. doi:10.1016/j.jadohealth.2013.11.021 PMC3999409 24759443

[4] Gershon P, Sita KR, Zhu C, . Distracted driving, visual inattention, and crash risk among teenage drivers. Am J Prev Med. 2019;56(4):494-500. doi:10.1016/j.amepre.2018.11.024 30799162

[5] The Zebra. The state of auto insurance in 2020. Accessed January 31, 2021. https://www.thezebra.com/state-of-insurance/auto/2020/

[6] Arumugam S, Bhargavi R. A survey on driving behavior analysis in usage based insurance using big data. J Big Data. 2019;6(1):1-21. doi:10.1186/s40537-019-0249-5

[7] Tselentis DI, Yannis G, Vlahogianni EI. Innovative motor insurance schemes: a review of current practices and emerging challenges. Accid Anal Prev. 2017;98:139-148. doi:10.1016/j.aap.2016.10.006 27723515

[8] Kyle Stock. Insurers know exactly how often American drivers touch their phones. Bloomberg. April 29, 2019. Accessed January 31, 2021. https://www.bloomberg.com/news/articles/2019-04-29/insurers-know-exactly-how-often-american-drivers-touch-their-phones

[9] Fortune Business Insights. Automotive usage based insurance market size, share & COVID-19 impact analysis, 2023-2030. Accessed December 17, 2023. https://www.fortunebusinessinsights.com/automotive-usage-based-insurance-market-104103.

[10] Delgado MK, Wanner KJ, McDonald C. Adolescent cellphone use while driving: an overview of the literature and promising future directions for prevention. Media Commun. 2016;4(3):79-89. doi:10.17645/mac.v4i3.536 27695663 PMC5041591

[11] Delgado MK, McDonald CC, Winston FK, . Attitudes on technological, social, and behavioral economic strategies to reduce cellphone use among teens while driving. Traffic Inj Prev. 2018;19(6):569-576. doi:10.1080/15389588.2018.1458100 29652523 PMC6215497

[12] Papadimitriou E, Argyropoulou A, Tselentis DI, Yannis G. Analysis of driver behaviour through smartphone data: The case of mobile phone use while driving. Saf Sci. 2019;119:91-97. doi:10.1016/j.ssci.2019.05.059

[13] Svenson O. Are we all less risky and more skillful than our fellow drivers? Acta Psychol (Amst). 1981;47(2):143-148. doi:10.1016/0001-6918(81)90005-6

[14] Bayer JB, Campbell SW. Texting while driving on automatic: considering the frequency-independent side of habit. Comput Human Behav. 2012;28(6):2083-2090. doi:10.1016/j.chb.2012.06.012

[15] Hayashi Y, Miller K, Foreman AM, Wirth O. A behavioral economic analysis of texting while driving: delay discounting processes. Accid Anal Prev. 2016;97:132-140. doi:10.1016/j.aap.2016.08.028 27614547 PMC5154926

[16] Kahneman D, Knetsch JL, Thaler RH. Anomalies: the endowment effect, loss aversion, and status quo bias. J Econ Perspect. 1991;5(1):193-206. doi:10.1257/jep.5.1.193

[17] Zeelenberg M, Beattie J, Van der Pligt J, De Vries NK. Consequences of regret aversion: effects of expected feedback on risky decision making. Organ Behav Hum Decis Process. 1996;65(2):148-158. doi:10.1006/obhd.1996.0013

[18] Dai H, Milkman KL, Riis J. The fresh start effect: temporal landmarks motivate aspirational behavior. Manage Sci. 2014;60(10):2563-2582. doi:10.1287/mnsc.2014.1901

[19] Duckworth AL, Gendler TS, Gross JJ. Situational strategies for self-control. Perspect Psychol Sci. 2016;11(1):35-55. doi:10.1177/1745691615623247 26817725 PMC4736542

[20] Papke LE, Wooldridge JM. Econometric methods for fractional response variables with an application to 401 (k) plan participation rates. J Appl Econ. 1996;11(6):619-632. doi:10.1002/(SICI)1099-1255(199611)11:6<619::AID-JAE418>3.0.CO;2-1

[21] Gross A. Think you’re in your car more? you’re right. Americans spend 70 billion hours behind the wheel. 2019. Accessed November 4, 2021. https://newsroom.aaa.com/2019/02/think-youre-in-your-car-more-youre-right-americans-spend-70-billion-hours-behind-the-wheel

