# Supplementary material for: Feedback and Financial Incentives for Reducing Cell Phone Use While Driving: A Randomized Clinical Trial
Source: JAMA Netw Open. 2024 Jul 10;7(7):e2420218. doi: 10.1001/jamanetworkopen.2024.20218 (PMC11238027; doi:10.1001/jamanetworkopen.2024.20218)
Supplement: Supplement 1. — Trial Protocol and Statistical Analysis Plan [file jamanetwopen-e2420218-s001.pdf]

Comparative Effectiveness of Financial Incentives and Nudges to Reduce Cellphone Use While Driving Among UBI Auto Policy Holders

Acronym: Progressive-UPenn-TrueMotion Investigation into Telephone Distraction of Operators With Nudges (PUT IT DOWN)

CLINICAL TRIAL PROTOCOL

Version 1.0
5/7/19

ClinialTrials.Gov Identifier:
NCT03833219

Principal Investigator:
M Kit Delgado, MD, MS
Assistant Professor of Emergency Medicine, University of Pennsylvania

Co-Investigators:
University of Pennsylvania:
Ian Barnett, PhD
Scott D. Halpern, MD, PhD
Jessica Hemmons, MS
Dina Abdel-Rahman, BS
Catherine McDonald, PhD, RN
Roy Rosin, MBA
Dylan Small, PhD
Kevin Volpp, MD, PhD
Douglas Wiebe, PhD
Flaura K. Winston, MD, PhD
Aria Xiong, MS

Progressive Insurance:
William Everett
Kristen Gaba

Truemotion, Inc.
Rafi Feingold
Ben Kotrc
Emma Radford

Contact Address:
Blockley Hall, 936
423 Guardian Drive
Philadelphia, PA 19104

Phone:
215-746-8256

Email:
Kit.Delgado@uphs.upenn.edu

Funded By:
Federal Highway Administration (FHWA), US Department of Transportation

Table of Contents

Trial Overview

1. Background and Significance
    1.1. Scope of Problem
    1.2. Existing Interventions
    1.3. Behavioral Drivers of Vehicle Safety
    1.4. Rationale for behavioral and financial interventions
2. TRIAL DESIGN
    2.1. Overview
    2.2. Setting
    2.3. Inclusion Criteria
    2.4. Participant Identification
    2.5. Consent Procedures
    2.6. Contact Information
    2.7. Baseline
    2.8. Baseline Assessment
    2.9. Allocation to Treatment
    2.10. Behavioral Interventions
        2.10.1. Intervention Arms
        2.10.2. Exit Survey
    2.11. Assessment Controls
    2.12. Outcomes Assessment
    2.13. Compensation
    2.14. Regulatory
    2.15. Contingencies and Participant Withdrawal
    2.16. Study Flow Diagram
3. OUTCOMES
    3.1. Definitions
        3.1.1. Primary Outcome
        3.1.2. Secondary Outcome 1
        3.1.3. Secondary Outcome 2
        3.1.4. Secondary Outcome 3
4. SAMPLE SIZE CALCULTION AND STATISTICAL ANALYSIS PLAN
    4.1. Analytic Methods
    4.2. Sample Size, Power, and Minimum Detectable Differences
    4.3. Subgroup Analyses and Effect Modification
5. DATA COLLECTION AND MANAGEMENT
    5.1. Data Collection Process
    5.2. Variables
    5.3. Data Quality and Validity
    5.4. Data Storage and Security
6. HUMAN SUBJECTS
    6.1. Risks to Human Subjects
    6.2. Adequacy of Protection Against Risk
        6.2.1. Recruitment and Informed Consent
        6.2.2. Protection against Risk
        6.2.3. Potential Benefits to Individual Participants
7. MONITORING
    7.1. Institutional Review Board (IRB)
    7.2. Data Safety and Monitoring

8. TIMELINE AND ENROLLMENT
  8.1. Timeline
  8.2. Screening and Enrollment
9. FUNDING
10. PUBLICATIONS
11. DATA SHARING
12. TASKS AND RESPONSIBILITIES
  12.1. Principal Investigator
  12.2. Co-Investigators
13. REFERENCES

Appendices:

I: Trial Variable Table

II: Intake Survey

III: Exit Survey

TRIAL OVERVIEW Title

Comparative Effectiveness of Financial Incentives and Nudges to Reduce Cellphone Use While Driving Among UBI Auto Policy Holders (PUT-IT-DOWN Study)

Clinical Trials Number

NCT03833219

Sources of monetary or material support

FHWA

Study Sites

National cohort of Progressive Snapshot Mobile auto insurance policy holders

Conditions studied

Distracted driving

Interventions

Push notification reminders, social comparison feedback, and financial incentives

Comparator

Self-monitoring (control)

Inclusion criteria

Progressive Snapshot Users with policy activated within recruitment period

Progressive Snapshot policy holder in one of the following states and regions: MI, PA, TX, FL, TN, OR, GA, NE, OK, OH, MT, MO, NV, CT, WI, MD, KY, MN, NH, NJ, AZ, ME, LA, SC, CO, MS, IN, IA, AL, ND, UT, RI, WV, WY, IL, AR, DE, KS, SD, NM, VT, ID, and the District of Columbia

Has an email address

Exclusion criteria

1. Progressive Snapshot Mobile App not updated to enable push notifications
2. Customer’s residential address is in a state in which phone use while driving is factored into insurance rating
3. Customer’s Snapshot Mobile App does not collect trip data with all sensors active
4. Customer in Snapshot program for < 30 days or more > 70 days

Study type

Interventional
Phase 1: Baseline – 30-70 days (usual care monitoring in Snapshot program)
• Phase 2: Intervention - 50 days – delivered to customers who agree to participate
• Phase 3: Post-intervention - remaining time until Snapshot rating complete (~30-60 days).

Randomization Arms

1. Control (monitor only)
2 Social comparison feedback
3. End of rating period incentive
4. End of rating period incentive + social comparison feedback
5. Weekly loss-framed incentive + social comparison feedback
6. Larger weekly loss-framed incentive + social comparison feedback

**Target sample size**	3000 Participants<br>500 participants per study arm.
**Primary outcome**	Percent of trip time engaged in active handheld phone use (also measured as seconds of handheld phone use while driving per hour of driving)

1. Background and Significance

1.1 Scope of Problem

The proportion of U.S. drivers witnessed visibly manipulating hand-held devices while driving has doubled since 2010 (NHTSA 2014), despite the fact that 97% of Americans know this is dangerous (AT&T 2012) and texting while driving is illegal in all but four states. Current policies to curb cellphone use while driving have had limited impact because they do not address the underlying impulsivity (Hayashi, 2015) that causes drivers to continue to engage in this behavior despite knowing it is against their best interests (Delgado 2016).

1.2 Existing Interventions

In November 2016, NHTSA released guidelines recommending the use of operating systems, aftermarket applications, and devices to enable a "driver mode" on smartphones to limit use while driving (Department of Transportation 2016). Akin to the federally mandated use of an "airplane mode" intended to limit cellular transmissions during air travel, a driver mode can be implemented on cellphones automatically limiting handheld cellphone use over certain speed thresholds consistent with driving based on native phone sensors or Bluetooth pairing with in-vehicle devices. These settings and applications have customizable configurations that can be programmed to activate when the car is moving, including locking the phone screen, silencing notifications, blocking incoming calls and text messages, and sending automated responses to incoming text messages. Research on these applications supports the efficacy of blocking technology on significantly reducing phone calls and text messaging while driving compared to control groups (Creaser et al. 2015; Ebel et al. 2015; Funkhouser and Sayer 2013). Third-party smartphone applications that enable a driver mode are also available for download ("Using Technology" 2014), but adoption and use have been limited. Though reasons for limited adoption have not been examined in the peer-review literature, media reports suggest that this is due to limited consumer acceptability (Richtel 2016). A recent study examining the adoption of similar smartphone apps to monitor and provide feedback on driving behavior among young drivers showed that perceived gains (e.g., financial incentives) and social norms are the biggest motivating factors in adoption of these applications. June 5, 2017, Apple (Cupertino, CA) announced a "do not disturb while driving mode" that became available as an iPhone operating system feature in Fall 2017 that can be turned on to lock the phone screen and silence and send automated responses to incoming text messages while driving (Sinder 2017).

Applications and settings aimed at limiting cellphone use while driving will only be effective from a population health standpoint if widely adopted and sustainably used. A promising approach to increased adoption of these applications and settings to sustainably change behavior is to increase perceived gains of these actions by providing financial incentives (Kervick et al. 2015; Musicant and Lotan 2016). This could be brought to scale through usage-based auto insurance programs that use mobile telematic smartphone apps to provide insurance discounts based on monitored driving and phone use behavior (Delgado et al. 2016). The effectiveness of financial incentive strategies in usage-based insurance programs could be enhanced by leveraging insights from behavioral economics, as has been effective for health behavior change (Delgado et al. 2016). This includes increasing the frequency and salience of incentives, to be more averse to losses than to gains. (Asch and Rosin 2016; Loewenstein et al. 2007, 2013). Despite the promise of redesigning usage based insurance programs with insights from behavioral economics, such strategies have not been tested previously.

1.3 Behavioral Drivers of Vehicle Safety

Conventional behavior change models assume individuals form behavioral change intentions based on the rational assessment of the costs and benefits and consistently act in their own interest. By contrast,

behavioral economics applies psychological insights to understand the many natural decisions people make that routinely deviate from their best interests. Through dozens of studies, we have demonstrated how a variety of common decision errors such as status quo bias, present bias (tendency to overweight immediate costs and benefits over future), loss aversion, regret aversion, and social norms, can be used to develop effective, high impact interventions for health behavior change. Our work has led to the development of a new health plan (Humana Simplicity), the enrollment process for automatic medication refills at CVS Health, financial incentives and smoking cessation programs among employees at General Electric and CVS. Further translating our deep expertise in behavioral economics to reducing cellphone use while driving could lead to a transformational paradigm shift in the approach to behavior change for this emerging cause of injury and risky driving in general.

1.4 Rationale for financial incentive and social comparison interventions

We have designed the interventions to be tested in our field experiment with several elements based on behavioral economic theory (Lowenstein, Volpp 2007), including: (1) promoting restraint from using a cellphone while driving by providing a substitute reinforcer in the form of incentives; (2) delivering incentives on a weekly basis to offset a heightened tendency to favor immediate gratification over delayed benefits among those who engage in texting while driving; (3) providing feedback via smartphones regarding accrued losses from non-adherence to maximize regret aversion; (4) framing reward-based incentives as losses to augment motivation through loss aversion; leveraging; and (5) social network theory and evidence that behavior change programs may be more effective when individuals are compared to those similar to them.

2. TRIAL DESIGN

2.1 Overview

We propose to conduct a randomized, control trial evaluating the comparative effectiveness of social comparison and financial incentive interventions with customers of Progressive's mobile phone usage-based insurance policy. A total of 3,000 adult participants will be enrolled with 500 participants per arm. We expect an attrition rate of 17% based on historical customer data.

2.2 Setting

The study will be conducted by Progressive Insurance and their mobile technology vendor TrueMotion. Progressive will be responsible for recruiting participants from their newly enrolled group of Snapshot customers. TrueMotion will be responsible for conducting study procedures including interventions, data collection, and participant payments. Penn researchers will be responsible for study design and data analysis.

2.3 Inclusion Criteria

Inclusion Criteria:

1. Progressive Snapshot Users with policy activated within recruitment period

2. Progressive Snapshot policy holder in one of the following states and regions: MI, PA, TX, FL, TN, OR, GA, NE, OK, OH, MT, MO, NV, CT, WI, MD, KY, MN, NH, NJ, AZ, ME, LA, SC, CO, MS, IN, IA, AL, ND, UT, RI, WV, WY, IL, AR, DE, KS, SD, NM, VT, ID, and the District of Columbia

3. Has an email address

Exclusion Criteria:

1. Progressive Snapshot Mobile App not updated to enable push notifications

2. Customer's residential address is in a state in which phone use while driving is factored into insurance rating

3. Customer's Snapshot Mobile App does not collect trip data with all sensors active

4. Customer in Snapshot program for < 30 days or more > 70 days

2.4 Participant Identification

Progressive will designate a cohort of customers eligible, based on inclusion and exclusion criteria, to invite to participate based on being in the Snapshot program on Day 30 to 70 at the date of study invitation.

2.5. Consent Procedures

Progressive Snapshot users will be sent an email from Progressive about participating in research. Those interested will follow a link that takes them to a website hosted by TrueMotion with information about participating in the study. Those that wish to participate will opt-in to enroll online through the website hosted by TrueMotion and complete an intake survey.

2.6. Contact Information

The research team and technology team at the University of Pennsylvania and TrueMotion (TMO), respectively, will not have access to participant information. Progressive will send invites with a Globally Unique Identifier (GUID) attached to the enrollment link. Progressive will send a list of GUIDs to TMO who will use these GUIDs to identify participants. TrueMotion will pair those GUIDs with participant emails that are given by the participant during the enrollment process. When participants enroll in the study, on the website hosted by TrueMotion, they will have to provide their e-mail address to receive their compensation. TrueMotion will use the email addresses provided by participants to compensate those that complete study surveys or are in a treatment arm that receives monetary incentives. Participants that have any issues or concerns during the study will be prompted to contact the study team via email at drivingstudy@gotruemotion.com.

2.7. Baseline

All new Progressive Snapshot customers will be monitored for 30-70 days during the baseline period. Progressive will designate a cohort of customers to invite into the study who will be on day 30-70 of their Snapshot monitoring period on the date of invitation. Study participants will be those that opt-in after the baseline period.

2.8. Baseline Assessment

Baseline Data

For Snapshot customers that opt-in to the study, Progressive will extract demographic data on the participant's age, marital status, gender, occupation, highest educational level, residential ZIP code, and urban, suburban, rural classification of the residential ZIP code (Kolko 2015).

Survey

After participants opt-in to the study, they will be prompted to complete an intake survey hosted by Truemotion. This will collect data on their vehicle characteristics (e.g. Bluetooth connectivity, GPS navigation), driving history (e.g. length of licensure), self-reported phone use while driving, perceptions of level phone use relative to others like them, use of settings to limit distracted driving (e.g. Do Not Disturb While Driving), and general behavioral characteristics (e.g. 5-trial delay discounting scale).

2.9. Allocation to Treatment

Progressive Snapshot users that opt-in to participate will be added to a study list tracked by TrueMotion. 3,000 participants have enrolled into the study, they will complete an intake survey, collecting information about driving history, demographics, and behavioral characteristics. Simple individual-level randomization will be used. TrueMotion will use a random number generator to randomize each user that opts in to 1 of the 6

arms: (Arm 1) control, (Arm 2) social comparison feedback, (Arm 3) end of rating period incentive, (Arm 4) end of rating period incentive and social comparison feedback (Arm 5) weekly loss framed incentive and social comparison feedback, (Arm 6) larger weekly loss framed incentive and social comparison feedback. Intervention will be delivered by TrueMotion through the Snapshot mobile app to the study participants. Additionally, iPhone users in all arms will get push notifications prompting them to activate the Do Not Disturb While Driving setting on iPhones.

2.10. Behavioral Interventions

2.10.1. Intervention Arms

**Intervention Arm**	**Notifications**	**Incentive**	
		**Timing**	**Size**
**Driver Mode Nudge (all arms, iPhone users only)**	3 times over 3 weeks: Prompt to enable Do Not Disturb While Driving. Linked to website with instructions and frequently asked questions: www.besafir.org/do-not-disturb.<br><br>Sample messages:<br>Arms 1-2: “Reduce annoying notifications when driving (while keeping the urgent ones!). Enable Do Not Disturb While Driving. Tap here to learn how!”<br><br>Arms 3-6: “Increase your chance to earn $ and reduce annoying notifications when driving (while keeping the urgent ones!). Enable Do Not Disturb While Driving. Tap here to learn how!”	N/A	N/A
**1. Control (monitor only)**	Continue in monitoring only mode	N/A	N/A
**2. Social comparison feedback**	**Initial push notification: Told they will be compared to drivers like them each week and that they will receive up to 3 stars.**<br><br>**Weekly push notification:**<br>**a.** Compare phone use % relative to Progressive Snapshot customers like them based (age, sex, marital status, urban/suburban/rural residence). Told can receive up to three stars.<br>• 1 star (if <50th percentile)<br>• 2 stars (if ≥ 50th percentile, <90th percentile)<br>• 3 stars if ≥ 90th percentile (top performer)<br><br>Sample message:<br>“⭐⭐ out of 3 stars. Almost there! You're better than at least half of our drivers at staying off your phone. A little less swiping, typing, and holding the phone and you'll be one of our best drivers! ”	N/A	N/A

**3. End of rating period incentive**	**Initial notification:**<br>a. Financial Incentive: Tell them they have a chance to win $50 in 50 days for keeping handheld phone use to a minimum.<br>• If <50th percentile), then paid $0<br>• If ≥ 50th percentile, <90th percentile), then paid $25<br>• If ≥ 90th percentile (top performer), then paid $50	Redeemed at end of rating period	Max: $50 ($1/day)
**4. End of rating period incentive + social comparison feedback**	**Initial notifications:**<br>**a.** Social comparison feedback: **Tell them they will be compared to drivers like them each week and that they will receive up to 3 stars.**<br><br>**b.** Financial Incentive: Tell them they have a chance to win $50 in 50 days for keeping handheld phone use to a minimum.<br>• If get 1 star (<50th percentile), then paid $0<br>• If get 2 stars (≥ 50th percentile, <90th percentile), then paid $25<br>• If get 3 stars ≥ 90th percentile [top performer]), then paid $50<br><br>**Weekly push notifications:**<br>**a. Social Comparison -** Compare to distribution for Progressive driver cohort (age, geographic area)<br>• 1 star (if <50th percentile)<br>• 2 stars (if ≥ 50th percentile, <90th percentile)<br>• 3 stars if > 90th percentile (top performer)<br><br>e.g. "⭐ out of 3 stars. Not bad, but more than half of our drivers used their phone less than you this week. Less swiping, typing, and holding your phone and you can move up the ranks!"<br><br>**b. Financial Incentive:** Reminder regarding the potential to earn incentive at end of intervention period.<br><br>e.g. "Remember, you need to use your phone less than half our other drivers to earn $ at the end of the study! Safety pays!"<br><br>**c. Post- trip 0 seconds distraction notification (throttled):** Each week, participant will receive a gain-framed message after the first driving trip with 0 seconds of phone use.<br><br>**e.**g. "Hey Now! You didn't swipe and type your phone while driving. Awesome! Keep up the	Redeemed at end of rating period	Max: $50 ($1/day)

	great work to stay safe and be one our best drivers!”		
**5. Weekly loss-framed incentive + social comparison feedback**	**Initial push notifications:**<br>a. Social comparison feedback: **Tell them they will be compared to drivers like them each week and that they will receive up to 3 stars.**<br><br>b. Financial incentive: Tell participants they have $1/day per coming to them over the next 50 days and that they will be paid each week. Told that they need to not use their phone while driving to keep from losing any money.<br><br>Weekly push notifications:<br>**a. Social Comparison -** Compare to distribution for Progressive driver cohort (age, geographic area)<br>1 star (if <50th percentile)<br>2 stars (if ≥ 50th percentile, <90th percentile)<br>3 stars if > 90th percentile (top performer)<br><br>Weekly push notifications:<br>**b. Social Comparison -** Compare to distribution for Progressive driver cohort (age, geographic area)<br>1 star (if <50th percentile)<br>2 stars (if ≥ 50th percentile, <90th percentile)<br>3 stars if > 90th percentile (top performer)<br><br>e.g. “⭐ out of 3 stars. Not bad, but more than half of our drivers used their phone less than you this week. Less swiping, typing, and holding your phone and you can move up the ranks!”<br><br>b. Financial incentive: Amount of incentive received this week + total amount of money lost during study = [MONEYLOST].<br><br>• If get 1 star (if <50th percentile), then paid $0<br>• If get 2 stars (if > 50th percentile, <90th percentile), then get paid $3.58<br>• If get 3 stars (if > 90th percentile [top performer]), then get paid $7.15<br><br>e.g. “You missed out on your weekly payment this week and a total of $[MONEYLOST] so far this study. No worries, fresh start! Use your phone less to get ⭐⭐ and $ next week! Safety pays!”	*Amount not lost for week delivered weekly*	Max: $50 ($1/day)

	**c. Post- trip 0 seconds distraction notification (throttled):** Each week, participant will receive a gain-framed message after the first driving trip with 0 seconds of phone use.<br>**e.**g. “Hey Now! You didn't swipe and type your phone while driving. Awesome! Keep up the great work to stay safe and be one our best drivers!”		
**6. Larger weekly loss-framed incentive + social comparison feedback**	**Initial push notifications:**<br>a. Social comparison feedback: **Tell them they will be compared to drivers like them each week and that they will receive up to 3 stars.**<br><br>**b.** Financial incentive: Tell participants they have $2/day per coming to them over the next 50 days and that they will be paid each week. Told that they need to not use their phone while driving to keep from losing any money.<br><br>Weekly push notifications:<br>**a. Social Comparison -** Compare to distribution for Progressive driver cohort (age, geographic area)<br>• 1 star (if <50th percentile)<br>• 2 stars (if ≥ 50th percentile, <90th percentile)<br>• 3 stars if > 90th percentile (top performer)<br><br>e.g. “⭐ out of 3 stars. Not bad, but more than half of our drivers used their phone less than you this week. Less swiping, typing, and holding your phone and you can move up the ranks!”<br><br>b. Financial incentive: Amount of incentive received this week + total amount of money lost during study = [MONEYLOST].<br>• If get 1 star (if <50th percentile), then paid $0<br>• If get 2 stars (if > 50th percentile, <90th percentile), then get paid $7.15<br>• If get 3 stars (if > 90th percentile [top performer]), then get paid $14.29<br><br>e.g. “You missed out on your weekly payment this week and a total of $[MONEYLOST] so far this study. No worries, fresh start! Use your phone less to get ⭐⭐ and $ next week! Safety pays!”	*Amount not lost for week delivered weekly*	Max: $100 ($2/day)

	**c. Post- trip 0 seconds distraction notification (throttled):** Each week, participant will receive a gain-framed message after the first driving trip with 0 seconds of phone use.<br>**e.**g. “Hey Now! You didn't swipe and type your phone while driving. Awesome! Keep up the great work to stay safe and be one our best drivers!”		

2.10.2 Exit Survey

Using Study ID#, Progressive will send an email to study participants with a link to an exit survey hosted by TrueMotion approximately 3-7 days before the end of the intervention period. This survey will elicit feedback from participants about their experience in the study and how the intervention impacted their behavior surrounding phone use while driving.

2.11. Assessment Controls

Participants allocated to the control arm that have iPhones will receive push notifications prompting them to enable Do Not Disturb While Driving once a week for 3 weeks, without receiving any feedback or goal support for the intervention period, 50 days.

2.12. Outcomes Assessment and Blinding

The primary outcome will be seconds of active handheld, non-call use per hour of driving. Research associates and investigators will be blinded to the allocation until outcome evaluation. It is not possible to blind subjects given the nature of the intervention. We do not expect any scenarios where emergency unblinding will be necessary.

2.13. Compensation

Participants will be compensated $10 for completing the intake survey and $10 for completing the End of Study Survey. Arms 3-6 will receive compensation based on their driving scores. Participants will be scored based on historical demographic data that has been split into 3 buckets: 1) low performers (<50%); 2) average performs 50-89%; 3) top performers >90%.

2.14. Regulatory

This study is IRB exempt. The Penn research team will not receive any PHI or identifiable information and all trial procedures and participant payments will be conducted and dispersed by TrueMotion. The trial is minimal risk to participants, given that the only possible risk will be from potential breach of confidentiality. TrueMotion will not have identifiable information of participants other than email address for communication and study payments. Regarding user driving data, Progressive will send a global unique identifier (GUID) to TrueMotion. TrueMotion will pair those GUIDs with those that have enrolled into the study. This way, TrueMotion will not have name/demographic data of participants connected with their Progressive Snapshot driving data. TrueMotion will send the list of enrolled GUIDS to Progressive. Only Progressive will have demographic information regarding which of their participants have enrolled into the study. Participants will voluntary provide their email address to TrueMotion to receive compensation.

The study has been registered on clinicaltrials.gov under NCT03833219

2.15. Contingencies and Participant Withdrawal

If a participant withdraws from the study, further communication will be stopped. Data collected prior to withdrawal will be maintained but additional data will not be collected.

2.16. Study Flow Diagram

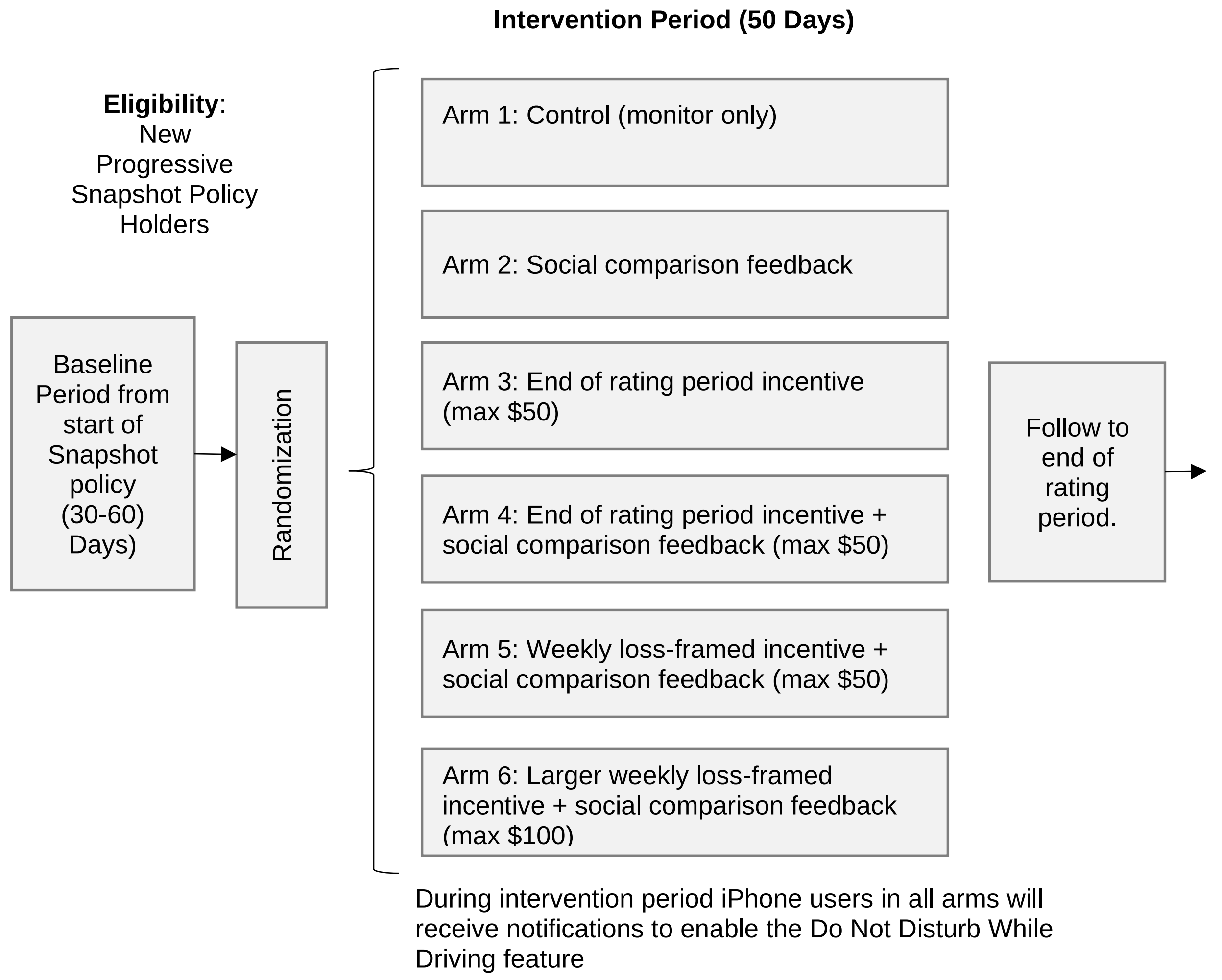

3. OUTCOMES

3.1. Definitions

3.1.1. Primary Outcome

Seconds of active handheld phone use per hour of driving. This is a composite outcomes that measures the proportion of total trip time in which the customer is engaged in handheld phone call use or non-call handheld use (e.g. texting, swiping, and typing) as measured by the Snapshot mobile application. Several studies (e.g. Klauer, *NEJM*, 2014) have demonstrated the association between handheld phone use, such reaching for phone, typing, swiping, dialing, and increased crash risk. This outcome is also known as the **active phone use percentage**. Passive phone use (e.g. phone is streaming GPS navigation directions or music without any typing, swiping, and handheld holding of the phone) is not included in this outcome. Customers are able to view their active phone use percentage for each trip in the Snapshot Mobile app. Trips are only counted if the Snapshot Mobile app predicts the customer was a driver for the trip based on the phone sensor data. In the Snapshot Mobile program, customers can open the Snapshot app and reclassify trips from driver trips to passenger trips and vice versa for up to 5 days. This outcome is currently not used to calculate future auto-insurance rates and discounts among eligible study participants.

3.1.2. Secondary Outcomes - Effectiveness

Seconds of active handheld call use per hour of driving. We will analyze the components of the primary outcome separately.

Seconds of active handheld non-call use per hour of driving. We will analyze the components of the primary outcome separately.

Hard breaking events per 100 miles. This is measured by the Snapshot Mobile app, reported for each trip, and used to calculate future auto-insurance rates and discounters.

Fast acceleration events per 100 miles. This is measured by the Snapshot Mobile app, reported for each trip, and used to calculate future auto-insurance rates and discounts.

3.1.3. Secondary Outcome 2 – Cost Effectiveness

Incremental Cost-Effectiveness Ratio. We will measure the incremental cost-effectiveness of the intervention arms relative to the control arm and to each other. This will be done by calculating incremental cost-effectiveness ratios as follows:

[(Incentive Costs in Intervention Arm) – (Incentive Costs in Comparison Arm)] / [(Seconds of active handheld use / hour of driving in Intervention Arm) – (Seconds of active handheld use / hour of driving in Comparison Arm)]

3.1.4. Secondary Outcome 3 - Acceptability

Acceptability of interventions will be measured using a mixed-methods approach:

Snapshot Mobile app notifications disabled. Given the eligible participants will have notifications enabled, if a customer disables notifications, this could indicate a lack of acceptability of push notification interventions.

Net promoter score. In the exit survey, participants will be asked **"***How likely is it that you would recommend the intervention you received to a friend or colleague?*" on a 0 to 10 scale.

Responses to open-ended questions in exit survey. Participants will be asked the following two open-ended questions: 1) "Please tell us what you *liked* about participating in this study;" 2) "Please tell us what *didn't like* about participating in this study and how we should improve the experience in the future." These qualitative responses will be coded for content related to intervention acceptability and emerging themes will be identified by randomization arm.

4. STATISTICAL ANALYSIS PLAN

4.1. Analytic Methods

The primary outcome is **seconds of active handheld phone use per hour of driving,** which can also be expressed as active phone use %. We will use logistic regression as our primary analytic model to assess effectiveness between and among the 5 interventions. Specifically, we will evaluate these 10 predefined contrasts:

1. Social Comparison Feedback versus Control (monitor only)
2. End of Rating Period Incentive versus Control (monitor only)
3. End of Rating period Incentive + Social Comparison Feedback versus Control (monitor only)
4. Weekly Loss-Framed Incentive + Social Comparison Feedback versus Control (monitor only)
5. Larger Weekly Loss-Framed Incentive + Social Comparison Feedback versus Control (monitor only)
6. End of Rating Period Incentive versus Social Comparison Feedback
7. End of Rating Period Incentive + Social Comparison Feedback versus Social Comparison Feedback
8. End of Rating Period Incentive + Social Comparison Feedback versus End of Rating Period Incentive
9. Weekly Loss-Framed Incentive + Social Comparison Feedback versus End of Rating Period Incentive + Social Comparison Feedback

10. Larger Weekly Loss-Framed Incentive + Social Comparison Feedback versus Weekly Loss-Framed Incentive + Social Comparison Feedback

Although longitudinal models such as generalized estimating equations might be considered in light of the repeated measures among individual participants, logistic regression is preferred in this case because the primary outcome collapses to a number between 0 and 1. The model will be adjusted for: 1) length of time in the Snaphshot program program during the Baseline period; 2) and observed primary outcome during the Baseline period.

Due to the multiple pre-planned contrasts, we will adjust for multiple comparisons using the Holm method. First, the 10 contrasts will be ranked (lowest to highest) based on the raw p-value from the logistic regression. For a contrast to be significant using the Holm threshold, the raw p-value must be below the threshold for each row calculated as (0.05 / [n remaining constrasts]). Correspondingly, for clarity of presentation, adjusted p values will be reported by multiplying the raw p value by the number of remaining contrasts. For example, the smallest p-value is multiplied by 10 to obtain the adjusted p-value.

4.2. Sample Size, Power, and Minimum Detectable Differences

To detect a clinically meaningful reduction of 60 seconds/hour of driving (i.e. an absolute reduction in phone use % of 1.67%), the minimum sample size is 2,406 (401 per arm).

With a target sample size of 3,000 and power of 0.80, we should be able to detect a difference between treatment arms of 52 seconds/hour of driving (i.e. an absolute reduction in phone use % of 1.44%). We would have a power of 0.94 to detect a difference of 60 seconds/hour of driving.

These estimates should be conservative as adding the rich set of baseline covariates (e.g. demographics, Apple car play, etc) to the model should increase the precision of the model estimates.

4.3. Subgroup Analyses and Effect Modification

For the 10 pre-defined contrasts, we will also assess the effect modification of having an iPhone versus Android phone. This will enable us to estimate the effectiveness of push notifications to enable automated activation of Do Not Disturb While Driving. These notifications only get sent to iPhones.

5. DATA COLLECTION AND MANAGEMENT

5.1. Data Collection Process

All study data, including survey data and driving data will be the collected by TrueMotion and demographic data will be collected by Progressive. All baseline and outcome variables will be obtained prospectively from elective surveys or through the Snapshot App maintained by TrueMotion. Penn researchers will not collect any study data and will receive de-identified data to analyze outcomes.

5.2. Variables

Measure library is included in the Appendix

5.3. Data Quality and Validity

Data quality and validity will be optimized by using a detailed data dictionary which will be distributed to TrueMotion. Data quality will be monitored by the TrueMotion development and tech team.

5.4 Transfer of Data

Study data, including survey data and driving data will be de-identified (email address removed) and transferred to the UPenn research via an encrypted .csv file uploaded to a secure, cloud based data storage

system hosted on PMACs servers. The file will be password protected and the password will be provided to the research team via telephone.

Demographic data from Progressive will be transferred in the same manner.

Demographic data will be matched with study data using a study ID # assigned to each participant.

5.5. Data Storage and Security

Any datasets and computer files are referred to by study ID. The study ID is also used on all analytical files. The Penn Medicine Academic Computing Services (PMACS) will store all data securely sent to the Penn research team. The data center is housed in Information Systems and Computing at 3401 Walnut Street. All data are stored in a single relational database, allowing researchers to correct mistakes. The same unique study ID is used to link these outside data to the participants. All data for this project will be stored on the secure/firewalled servers of the PMACS Data Center, in data files that will be protected by multiple password layers. These data servers are maintained in a guarded facility behind several locked doors, with very limited physical access rights. They are also cyber-protected by extensive firewalls and multiple layers of communication encryption. Electronic access rights are carefully controlled by UPenn system managers. This multi-layer system of data security, identical to the system protecting the University of Pennsylvania Health Systems medical records, greatly minimizes the risk of loss of privacy.

6. HUMAN SUBJECTS

6.1. Risks to Human Subjects

This project proposes minimal risk. There is a risk of breach of confidentiality for participants receiving study information from TrueMotion. For the Penn research team project is primarily a data assessment of an existing dataset, and all information used was gathered previously by Progressive and TrueMotion and is de-identified.

6.2. Adequacy of Protection Against Risk

Participants are Progressive Insurance Snapshot® customers that have the Snapshot® app downloaded onto their mobile devices. Customers have agreed to have data sharing and location services turned on. These users have previously agreed to Snapshot® terms of service (https://www.progressive.com/support/legal/snapshot-app-terms-conditions/?theme=neutral) and privacy statement (https://www.progressive.com/support/legal/snapshot-privacy-statement/). Progressive will assign a Research ID to each Study Participant. Progressive and TrueMotion will not disclose to the University the key for the association of Study Participant to Research ID. Data will be transferred between research partners using encryption and password protection, as described in section 5.4.

The University of Pennsylvania research team will not have access or hold any PHI for any of these users. All user names have been de-identified. Prior to sharing the data with us TrueMotion will remove identifiers (email address).

6.2.1. Recruitment and Informed Consent

Progressive will designate a cohort of customers to invite to study on Day 30 to 70 of their Snapshot Mobile enrollment. This will be the “Eligible Cohort.”

Progressive will send an email to a subset of its customers who use the App with an invitation to participate in the Research by providing their express consent after the baseline period. The email will contain a link to a TrueMotion landing page specific to the Research and will contain a GUID. Once such a Progressive customer consents to participate, that customer will be considered a Study Participant for purposes of this study, until such time as a Study Participant elects to opt-out of participation. Progressive will assign the Study ID to each Study Participant. TrueMotion will provide Progressive with the GUID for each Study Participant and Progressive will then provide to TrueMotion the Study ID associated with such GUID.

Those interested will follow a link that takes them to a website hosted by TrueMotion with information about participating in the study. Those that wish to participate will opt-in to enroll online through the website hosted by TrueMotion.

6.2.2. Protection against Risk

In order to protect study participants form potential risks related to the loss of confidentiality and due to any discomfort that they may experience in answering any questions, the following steps will be taken: 1) Participants will be told that they can withdraw from the study at any time by contacting us. 2) All information provided by the participant will be referenced to a subject ID#. The participant's ID# can only be connected to their identifiable information only through GUID mapping file held only by Progressive. The Penn research team will have no access to PHI. All data and files will be entered on computers protected by passwords and stored in a locked office. 3) All research staff will be trained in the importance of maintaining confidentiality and will undergo annual mandatory training of human subjects' research. 4) All participant data will be presented in aggregate and no individuals will be identified individually.

6.2.3. Potential Benefits to Individual Participants

This information could allow the research team and Progressive to gain insight about effective usage based insurance (UBI) program strategies for reducing distraction from phone use while driving.

7. MONITORING

7.1. Institutional Review Board (IRB)

This study has been approved as an IRB exempt protocol.

7.2. Data Safety and Monitoring

The limited data collected by TrueMotion through the Snapshot app and will be de-identified and manually uploaded by members of the TrueMotion team through secure .CSV files. Data will not be analyzed in real time. It will be securely stored on the study investigator's computers on a Penn Medicine secure network server using password protection and encryption. All data received will be de-identified and will remain de-identified. All data obtained in the study will be used exclusively for the purposes of the proposed research. Users of this platform have been assigned a Study ID# and no identifiers will be contained in this file. This data will be linked by a programmer utilizing coded study IDs and operating behind a University firewall. The dataset is blinded of all personally identifiable information. (Variables in Appendix)

8. TIMELINE AND ENROLLMENT

8.1. Timeline

Study period is approximately 150 days.

- Phase 1: Baseline - 30 to 70 days (usual care monitoring in Snapshot program)
- Phase 2: Intervention - 50 days – delivered to opt-in Participants
- Phase 3: Post-intervention - remaining time until Snapshot rating complete (~30-60 days).

8.2. Screening and Enrollment

Progressive will designate a cohort of customers to invite to study on Day 30-70 of their Snapshot Mobile enrollment. This will be the "Eligible Cohort."

Progressive Snapshot users will be sent an email from Progressive about participating in research between day 30-70 of baseline. Those interested will follow a link that takes them to a website hosted by TrueMotion with information about participating in the study. Those that wish to participate will opt-in to enroll online through the website hosted by TrueMotion.

Once the participant is randomized, they will receive an email about their arm assignment. The email will provide details about the study (i.e. “What do I need to do?” and “How will I get paid?”) and contact information for any questions.

Once all 3,000 participants have been enrolled, the intervention period will begin (up to 2 weeks after enrollment).

9. FUNDING

Funding for the present trial is provided by FHWA. The funding agencies have no role in the design and conduct of the study, collection, management, analysis, and interpretation of the data, preparation, review, or approval of the manuscript, or the decision to submit the manuscript for publication.

10. PUBLICATIONS

We plan to publish the findings in conference proceedings and/or peer-reviewed journals.

11. DATA SHARING

The investigator has a collaborative research agreement between Progressive, TrueMotion, and recipient researchers (University of Pennsylvania research staff) before sharing a limited dataset (driving trip summaries, phone usage, rating, etc.).

12. TASKS AND RESPONSIBILITIES

<u>Principal investigator:</u> Overall responsibility for protocol development, intervention development, budget overview, data dictionary development, ethical approval, trial registration, trial oversight, and the data and safety monitoring board, assessment of overall recruitments, potential, data analysis, and dissemination and presentation of results.

<u>Co-Investigators:</u> Also protocol development, data dictionary development, trial oversight, dissemination of results.

12.1. Principal Investigator

Mucio Kit Delgado, MD, MS

12.2. Co-Investigators

University of Pennsylvania:
Ian Barnett, PhD
Scott D. Halpern, MD, PhD
Jessica Hemmons, MS
Dina Abdel-Rahman, BS
Catherine McDonald, PhD, RN
Roy Rosin, MBA
Dylan Small, PhD
Kevin Volpp, MD, PhD
Douglas Wiebe, PhD
Flaura K. Winston, MD, PhD
Aria Xiong, MS

Progressive Insurance:
William Everett
Kristen Gaba

Truemotion, Inc.
Rafi Feingold
Ben Kotrc
Emma Radford

13. REFERENCES

Asch DA, Rosin R. Engineering social incentives for health. *N Engl J Med*. 2016;375:2511–2513.

AT&T. AT&T Teen Driver Survey: Executive Summary 2012. https://www.att.com/Common/aboutus/txtingdriving/attteensurveyexecutive.pdf

Creaser JI, Edwards CJ, Morris NL, Donath M. Are cellular phone blocking applications effective for novice teen drivers? *J Safety Res*. 2015;54: 75–78.

Delgado MK, Wanner KJ, McDonald C. Adolescent cellphone use while driving: an overview of the literature and promising future directions for prevention. *Media Commun*. 2016;4(3):79–89.

Department of Transportation, NHTSA. Visual–Manual NHTSA Driver Distraction Guidelines for Portable and Aftermarket Devices. 2016. Docket No. NHTSA-2013-0137. Available at: https://www.nhtsa.gov/press-releases/us-dot-proposes-guidelines- address-driver-distraction-caused-mobile-devices-vehicles.

Ebel B, Boyle L, O'Connor S, et al. Randomized Trial of Cell Phone Blocking and In-Vehicle Camera To Reduce High-Risk Driving Events among Novice Drivers. San Diego, CA: Pediatric Academic Societies; 2015.

Funkhouser D, Sayer JR. *Cellphone Filter/Blocker Techonology Field Test*. Washington, DC: NHTSA; 2013. Report No. HS 811 863.

Hayashi Y, Russo CT, Wirth O. Texting while driving as impulsive choice: a behavioral economic analysis. Accident Analysis & Prevention. 2015;83:182-189.

Holm S. A simple sequentially rejective multiple test procedure. Scandinavian Journal of Statistics 1979 6:65-70.

Kervick AA, Hogan MJ, O'Hora D, Sarma KM. Testing a structural model of young driver willingness to uptake smartphone driver support systems. *Accid Anal Prev*. 2015;83:171–181.

Klauer SG, Guo F, Simons-Morton BG, Ouimet MC, Lee SE, Dingus TA. Distracted driving and risk of road crashes among novice and experienced drivers. New England journal of medicine. 2014; 370(1)54-9.

Kolko, Jed. "How Suburban Are Big American Cities?" FiveThirtyEight, 15 May 2015, fivethirtyeight.com/features/how-suburban-are-big-american-cities/.

Loewenstein G, Asch D, Volpp K. Behavioral economics holds potential to deliver better results for patients, insurers, and employers. *Health Aff (Millwood)*. 2013;32:1244–1250.

Loewenstein G, Brennan T, Volpp KG. Asymmetric paternalism to improve health behaviors. *JAMA*. 2007;298:2415–2417.

Musicant O, Lotan T. Can novice drivers be motivated to use a smartphone based app that monitors their behavior? *Transp Res Part F Traffic Psy- chol Behav*. 2016;42:544–557.

National Highway Traffic Safety Administration. Distracted Driving 2014. Traffic Safety Facts Research Note. Available at: http://www.-nrd.nhtsa.dot.gov/CATS/index.aspx

Richtel M. Phone makers could cut off drivers. So why don't they? *New York Times*. September 24, 2016.

Sinder M. iPhone will get "Do Not Disturb While Driving" mode in iOS 11 this fall. *USA Today*. June 5, 2017.

Using Technology to Fix the Texting-While-Driving Problem. *National Public Radio*. April 24, 2014.

Appendix I

Data to be transferred to UPenn for Participants (Baseline, Intervention, and Post-Intervention Phases).	
	Variables
Progressive Demographic Data	
	Research ID
	Age
	Sex
	Marital status
	Residential ZIP code
Progressive data for each trip	
	Research ID
	Trip date
	Trip start time
	Trip end time
	Total trip time
	Seconds of phone use (total)
	Seconds of handheld phone calls
	Seconds of handsfree calls
	Seconds of active non-call phone use
	Seconds of passive non-call phone use
	Number of handheld phone use events
	Number of hard breaking events
	Number of fast acceleration events
	Randomization arm (aka test cell)
	Study phase (baseline vs. intervention vs. post- intervention
Phone System and Attrition data collected by TrueMotion	
	Research ID
	Phone operating system (iPhone vs. Android)
	Date of App uninstall
	Date Notifications turned off
Data collected via intake survey administered by TrueMotion	
	Research ID
	Responses to survey questions (see attached)

Appendix II

Intake Survey

Safe Driving Study

Safe Driving Study Survey Sign-up

Hello and thanks for checking out this exclusive opportunity! Progressive has teamed up with researchers at the University of Pennsylvania and TrueMotion, a mobile technology company, to test if the Snapshot Mobile app can help reduce distracted driving. Participating is easy and you can help us learn how to promote safe driving behaviors and earn money doing it!

What's in it for me?

- **You get a minimum of $20 in Amazon gift cards:**
- **o $10 for completing a 10-minute survey below**

  o $10 for completing another 10-minute survey just before the end of the study (in early June)

- **Plus, you will have a chance to get an additional$50 to $100 in Amazon gift cards based on what test group you're placed in and your driving behaviors!**
- **If you sign up, you may begin to receive notifications once in a while from the Snapshot Mobile app**

How does this work?

- **Any money earned in this study is not part of your Progressive auto policy or discount.**
- **All driving information collected during the course of this study will be from the Progressive Snapshot Mobile app and protected as described in the Progressive Snapshot Mobile privacy policy.**

Enrolling is easy!

- **If you agree to participate, you will be immediately redirected to complete our intake survey to get your first $10. The survey should take approximately 10 minutes to complete. We'll need your email address to send you any earned money.**
- **Feel free to email us at drivingstudy@gotruemotion.com if you have any questions about the study.**

* Do you agree to participate in this research study?

◯ Yes

◯ No

* Please enter the email address where you would like to receive your gift cards:

[ ]

Thanks for taking the time to complete this survey and providing us a little more information about yourself!

The survey may take about 10 minutes to complete. The information collected from you will help the research team develop ways to make our roads safer in the future.

After completing this survey, you will be emailed a $10 Amazon gift code, within 1 week, to the email address you just provided. You need to finish the survey to get the $10.

* Which type of smartphone do you use with Snapshot Mobile?

Apple iPhone Android

Smartphone

How often have you placed your phone in a mount or holder while driving in the past two weeks?

Never

Some trips

Most trips

How often have you connected your phone to your car's audio using Bluetooth or a USB cable while driving in the past two weeks?

Never

Some trips

Most trips

Does your car allow for your phone to connect to a dashboard touchscreen that allows you to control phone apps and functions (e.g. Apple CarPlay, Android Auto, Ford Sync, GM MyLink, Toyota Entune etc.)?

Yes

No

How often have you used the dashboard touchscreen instead of your phone screen to check text messages, control music, and use navigation apps while driving?

Never

Some trips

Most trips

How often have you let a passenger use your phone while you were driving in the last two weeks?

Never

1 to 2 days

3 days or more

How often have you ridden as a passenger in a car (including a rideshare or taxi) and used your phone in the last two weeks?

Never

1 to 2 days

3 days or more

Do you currently use Do Not Disturb While Driving or a similar phone setting that silences and hides notifications while driving?

Yes

No

All contacts

I do not know

Is Do Not Disturb While Driving or the similar setting set to come on automatically when you start driving or do you have to manually turn it on when you want to?

Yes - comes on automatically No -

have to turn it on manually

Does Do Not Disturb While Driving auto-respond to incoming text messages for your list of Favorite contacts or to anyone who sends you a text message?

No one (auto-reply is disabled)

Recents

Favorites

For the next few questions, think about how your driving behavior compares to others like you (similar age, gender, where you live..).

But first, some basic demographic questions. What's the highest level of education you have completed?

Less than a high school diploma

High school degree or equivalent (e.g. GED) Some

college, no degree

College degree

Some graduate-level courses, no degree

Post-graduate degree

What is your annual household income?

Under $20,000

$20,000 - $29,999

$30,000 - $39,999

$40,000 - $49,999

$50,000 - $59,999

$60,000 - $74,999

$75,000 - $99,999

$100,000 - $124,999

$125,000 - $149,999

Over $150,000

What is your race? (Select one or more responses)

White or Caucasian Black

or African American

American Indian or Alaska Native Asian or

Asian American

Native Hawaiian or other Pacific Islander Other

(please specify)

Are you Hispanic or Latino?

- Yes
- No

Compared to drivers like me, I think I use my phone while driving:

- More than others
- About the same as others
- Less than others

Compared to drivers like me, if I were to type a reply to a text message while driving, I think I would be:

- More likely to get into a crash than others
- About as likely to get into a crash as others
- Less likely to get into a crash than others

Compared to drivers like me, I think my rate of hard braking events is:

- More than others
- About the same as others
- Less than others

How many years have you had your driver's license?

- 0 to 4
- 5 to 9
- 10 to 14
- 15 or more

How many tickets for a traffic violation (like speeding or going through a red light) have you received in the last 5 years? Remember, this information is confidential and will not affect your insurance rating.

- ○ 0
- ○ 1
- ○ 2
- ○ 3 or more

How many car accidents have you been involved in as a driver in the last 5 years (whether your fault or not)? Remember, this information is confidential and will not affect your insurance rating.

0

1

2

3 or more

Think about the last 2 weeks. How often did you engage in the following behaviors while you were driving and the car was in motion (i.e. not stopped at a red light)?

	Never	Sometimes (some trips)	Often (most trips)
Talked on your phone while it was in your hand			
Talked on the phone using hands-free technology (like Bluetooth)			
Read text messages and app notifications (SMS, email, Snapchat, Facebook messenger, etc.) while your phone was in your hand			
Typed messages (SMS, email, Snapchat, Facebook messenger, etc.) while your phone was in your hand			
Sent text messages using hands-free technology			
Checked social media notifications (Facebook, Twitter, Instagram, etc.)			
Touched your phone to change the music			
Touched your phone to interact with a navigation app			

How often do you find yourself checking your phone to view a text message, email, or push notification while driving and not realize you are doing it?

- Never
- Rarely
- Sometimes
- Very Often
- Always

Do you find it hard to stop yourself from checking an incoming text message, email, or push notification while driving?

- Not at all
- Slightly
- Moderately
- Very
- Extremely

For the next few questions, think about how likely it would be for you to willingly give up the following behaviors while driving (again, while the car is in motion).

	Not willing to give up	Somewhat willing to give up	Willing to give up
Reading incoming texts			
Sending texts			
Reading or sending emails			
Making or receiving phone calls with the phone in your hand			
Using navigation apps such as Google or Apple Maps			
Using or checking/browsing social media apps like Instagram and Facebook			
Using/searching music apps such as Pandora or Spotify			

The next questions ask about behavior related to your mobile phone use. Read each statement and select how often each behavior describes you. Do Not spend too much time on each statement. Answer quickly and honestly.

	Strongly Agree	Agree	Neutral	Disagree	Strongly Disagree
I often think about my mobile phone when I am not using it					
I often use my mobile phone for no particular reason					
Arguments have arisen with others because of my mobile phone use					
I interrupt whatever else I am doing when I am contacted on my mobile phone					
I feel connected to others when I use my mobile phone					
I lose track of how much I am using my mobile phone					
The thought of being without my mobile phone makes me feel distressed					
I have been unable to reduce my mobile phone use					

We're almost done! The final few questions ask you to make decision. Don't think too long about each answer.

Which would you rather have?

$1000 in 3 weeks

$500 now

Which would you rather have?

$1000 in 2 years

$500 now

Which would you rather have?

$1000 in 1 day

$500 now

Which would you rather have?

$1000 in 8 years

$500 now

Which would you rather have?

$1000 in 4 months

$500 now

Which would you rather have?

$1000 in 4 days

$500 now

Which would you rather have?

$1000 in 4 hours

$500 now

Which would you rather have?

$1000 in 18 years

$500 now

Which would you rather have?

$1000 in 4 years

$500 now

Which would you rather have?

$1000 in 8 months

$500 now

Which would you rather have?

$1000 in 2 months

$500 now

Which would you rather have?

$1000 in 1.5 weeks

$500 now

Which would you rather have?

$1000 in 2 days

$500 now

Which would you rather have?

$1000 in 9 hours

$500 now

Which would you rather have?

$1000 in 2 hours

$500 now

Which would you rather have?

$1000 in 25 years

$500 now

Which would you rather have?

$1000 in 12 years

$500 now

Which would you rather have?

$1000 in 5 years

$500 now

Which would you rather have?

$1000 in 3 years

$500 now

Which would you rather have?

$1000 in 1 year

$500 now

Which would you rather have?

$1000 in 6 months

$500 now

Which would you rather have?

$1000 in 3 months

$500 now

Which would you rather have?

$1000 in 1 month

$500 now

Which would you rather have?

$1000 in 2 weeks

$500 now

Which would you rather have?

$1000 in 1 week

$500 now

Which would you rather have?

$1000 in 3 days

$500 now

Which would you rather have?

$1000 in 1.5 days

$500 now

Which would you rather have?

$1000 in 12 hours

$500 now

Which would you rather have?

$1000 in 6 hours

$500 now

Which would you rather have?

$1000 in 3 hours

$500 now

Which would you rather have?

$1000 in 1 hour

$500 now

End of the Survey

Thank you for completing this survey! You can expect a gift card in your email within 7 days.

Appendix III

Safe Driving Study Exit Survey

Thank you for taking the time to complete this survey, as the Safe Driving Study comes to an end this weekend. You should be able to complete this survey in less than 10 minutes.

The information collected from you will help the research team develop ways to make our roads safer in the future. After completing this survey, you will be emailed a $10 Amazon gift code. You'll be asked to provide your email address so you can receive this payment.

If you have any questions, please contact the research manager at the University of Pennsylvania at: drivingstudy@gotruemotion.com

* Email address for $10 gift code:

For the next few questions, think about how your driving behavior compares to others like you (similar age, gender, where you live..).

Compared to drivers like me, I think I use my cell phone while driving:

More than others

About the same as others Less

than others

Compared to drivers like me, if I were to type a reply to a text message on my phone while driving, I think I would be

More likely to get into a crash than others

About as likely to get into a crash as others

Less likely to get into a crash than others

Compared to drivers like me, I think my rate of hard braking is:

Better than others

About the same as others

Worse than others

Do you currently use Do Not Disturb or similar phone setting that silences and hides notifications while driving?

Yes

No

Is Do Not Disturb While Driving or the similar setting set to come on automatically when you start driving or do you have to manually turn it on when you want to?

Yes – comes on automatically No -

have to turn it on manually

Does Do Not Disturb While Driving auto-respond to incoming text messages for your list of Favorite contacts or to anyone who sends you a text message?

No one (auto reply is disabled)

Recents

Favorites

All contacts

Don’t know

What, if anything, caused to you reduce your handheld phone use while driving after enrolling in this study? (select all that apply)

Activating the Do Not Disturb While Driving setting or a similar setting

Tracking my own phone use while driving

Being more aware of how much I use my phone compared to drivers like me

The possibility of getting additional money for reducing my phone use while driving

Nothing caused me to reduce my phone use while driving

Other (please specify)

Which additional strategies did you adopt to help yourself use your phone less while driving? (Please check all that apply):

- [ ] Placing my phone out of reach Using
- [ ] more hands-free options Giving my
- [ ] phone to a passenger Turning my
- [ ] phone off
- [ ] Ignoring notifications
- [ ] Muting my phone None
- [ ] Other (please specify)
- [ ]

How likely is it that you would recommend this program to a friend or colleague?

NOT AT ALL LIKELY EXTREMELY LIKELY

0	1	2	3	4	5	6	7	8	9	10

Did the app have any technical issues? (Please check all that apply):

- [ ] No
- [ ] Registered passenger trips as driver trips Registered
- [ ] driver trips as passenger trips
- [ ] Phone use or driving measurements were inaccurate Other
- [ ] (please specify)

If an insurance company sent weekly push notification feedback on your driving behaviors would you opt-out?

- ○ Absolutely Not
- ○ Somewhat likely will not
- ○ Neutral
- ○ Somewhat Likely
- ○ Absolutely

If an insurance company sent weekly incentives based on your driving behaviors would you opt-out?

- ◯ Absolutely Not
- ◯ Somewhat likely will not
- ◯ Neutral
- ◯ Somewhat Likely
- ◯ Absolutely

Please tell us what factors *helped* you reduce the amount of time you used your phone while driving while participating in this study.

Please tell us what you *liked* about participating in this study.

Please tell us what you *didn't like* about participating in this study and how we should improve the experience in the future.

Please give us your suggestions for how we could best encourage drivers to be safer on the roads with apps like Snapshot mobile.

7/24/19 Summary of Modifications to Stastiscal Analysis Plan

The purpose of this modification is to clarify aspects of the statistical analysis plan prior to commencing the statistical analysis.

1. We clarified the primary analysis statistical model will be a factional regression with a logit link rather than a logistic regression model.
2. We pre-specified the covariates to be included in the primary analysis statistical model
3. We further clarified the variables to be considered as effect modifiers and delineated those that will be considered in the primary analysis with hypothesized directions and the others to be considered in a separate hypothesis generating secondary analysis
4. We described a mediation analysis of whether activating iPhone's Do Not Disturb While Driving during the trial mediates differences in the primary outcome by phone type.

4. STATISTICAL ANALYSIS PLAN

4.1. Analytic Methods

The primary outcome is **seconds of active handheld phone use per hour of driving,** which can also be expressed as active phone use %. We will use fractional regression with a logit link function as our primary analytic model to assess effectiveness between and among the 5 interventions. Specifically, we will evaluate these 10 predefined contrasts:

11. Social Comparison Feedback versus Control (monitor only)
12. End of Rating Period Incentive versus Control (monitor only)
13. End of Rating period Incentive + Social Comparison Feedback versus Control (monitor only)
14. Weekly Loss-Framed Incentive + Social Comparison Feedback versus Control (monitor only)
15. Larger Weekly Loss-Framed Incentive + Social Comparison Feedback versus Control (monitor only)
16. End of Rating Period Incentive versus Social Comparison Feedback
17. End of Rating Period Incentive + Social Comparison Feedback versus Social Comparison Feedback
18. End of Rating Period Incentive + Social Comparison Feedback versus End of Rating Period Incentive
19. Weekly Loss-Framed Incentive + Social Comparison Feedback versus End of Rating Period Incentive + Social Comparison Feedback
20. Larger Weekly Loss-Framed Incentive + Social Comparison Feedback versus Weekly Loss-Framed Incentive + Social Comparison Feedback

Although longitudinal models such as generalized estimating equations might be considered in light of the repeated trip measures among individual participants, fractional regression with a logit link is preferred in this case because the primary outcome collapses to a proportion between 0 and 1 that is weighted by travel time within and across trips. Furthermore, weekly feedback messages and incentives are delivered based on the weekly averages of seconds of active handheld phone use time divided by weekly total trip time. The model will be adjusted for: 1) length of time in the Snaphshot program during the Baseline period; 2) and observed

primary outcome during the Baseline period. Other covariates to be included in the model include:

Proposed baseline covariates to be included in primary outcome analysis model
1. Baseline period active phone use % (i.e. total active handheld phone use seconds / total trip seconds)
2. Proportion of period active phone use % due to handheld calls vs. handheld non-call use
3. Mean hours of driving/week during baseline period
4. Age
5. Sex
6. Marital status
7. Urban-suburban-rural residence
8. Residence in state with universal handheld ban
9. Race/ethnicity
10. Income
11. Education level
12. Phone type (iPhone vs. Android)
13. Baseline use of automated Do Not Disturb While Driving setting (Y/N)
14. Bluetooth/USB car connectivity (Y/N)
15. Dashboard touchscreen (Y/N)
16. Frequency of letting passenger use phone
17. Frequency of riding as a passenger
18. Number of prior traffic violations in prior 5 years
19. Number of car crashes in prior 5 years

Due to the multiple pre-planned contrasts, we will adjust for multiple comparisons using the Holm method. First, the 10 contrasts will be ranked (lowest to highest) based on the raw p-value from the logistic regression. For a contrast to be significant using the Holm threshold, the raw p-value must be below the threshold for each row calculated as (0.05 / [*n* remaining contrasts]). Correspondingly, for clarity of presentation, adjusted p values will be reported by multiplying the raw p value by the number of remaining contrasts. For example, the smallest p-value is multiplied by 10 to obtain the adjusted p-value.

4.2. Sample Size, Power, and Minimum Detectable Differences

To have power of 0.80 to detect a clinically meaningful reduction of a 2% difference in amount of trip time spent actively using a phone (72 seconds/hour of driving), the minimum sample size is 1,842 (301 per arm).

With a target sample size of 3,000 and power of 0.80, we should be able to detect a difference between treatment arms of 52 seconds/hour of driving (i.e. an absolute reduction in phone use % of 1.44%). We would have a power of 0.94 to detect a difference of 60 seconds/hour of driving.

These estimates should be conservative as adding the rich set of baseline covariates (e.g. demographics, Apple Car Play, etc) to the model should increase the precision of the model estimates.

4.3. Subgroup Analyses, Effect Modification, and Mediation Analysis

For the 10 pre-defined contrasts, we will also assess the effect modification of having an iPhone versus Android phone. This will enable us to estimate the effectiveness of push notifications to encourage the automated activation of Do Not Disturb While Driving. These notifications only get sent to iPhones. In the primary analysis, we will also determine the extent to which relative reductions in phone use are modified by the amount of baseline phone use.

**Proposed variables to be considered as effect modifiers in the primary analysis**
1. iPhone vs. Android. Hypothesis: the relative reductions in active handheld phone use will be greater among those with iPhones due to the notification to encourage Do Not Disturb While Driving.
2. Quartile of baseline period active phone use % (i.e. total active handheld phone use seconds / total trip seconds). Hypothesis: the relative reductions in active hand held phone use while be greater among those in the highest quartile of baseline phone use due to greatest opportunity for improvement and least opportunity for improvement in the lowest quartiles.
3. Proportion of period active phone use % due to handheld calls vs. handheld non-call use. Hypothesis: the relative reductions in active hand held phone use while be greater among those with a greater proportion of handheld phone use due handheld call use vs. non-call use due to multiple available technological means to switch to handsfree calls.
**Proposed variables to be considered as effect modifiers in hypothesis generating secondary analyses**
1. Baseline use of automated Do Not Disturb While Driving setting (Y/N)
2. Baseline use of a phone mount
3. Mean hours of driving/week during baseline period
4. Age
5. Sex
6. Marital status
7. Urban-suburban-rural residence
8. Residence in state with universal handheld ban
9. Income
10. Education level
11. Number of prior traffic violations in prior 5 years
12. Number of car crashes in prior 5 years
13. Self-reported frequency of types of handheld phone use while driving (e.g. phone call, text messaging, adjusting navigation app, changing music app, social media app)
14. Willingness to give up phone use while driving scale
15. Automaticity scale of phone use while driving
16. Compulsive phone use scale
17. Delay discounting rate

We will also conduct a mediation analysis to determine whether activation of an automated Do Not Disturb While Driving setting during the trial as measure on intake and exit surveys mediates the primary outcome by treatment arm and by phone type.

4.4 Sensitivity Analyses

The primary analysis will only include trip data classified as driver trips. It is possible that the interventions may cause customers to reclassify some driver trips to passenger trips within the allowable window of 5 days within the Snapshot Program. This may be done to: 1) appropriately to correct an incorrect classification by the Snapshot app; or 2) to inappropriately drop driver trips with high levels of phone distraction from being factored in the performance calculations in the intervention arms. Therefore we will assess the robustness of the primary results to the inclusion and exclusion of driver trips that participants reclassified to passenger trips in both the baseline and intervention periods.

4.5 Post-Intervention Period Analysis

The primary outcome analysis will be repeated for the post-intervention period to determine whether any behavior change is sustained after the interventions are discontinued.]
