# Supplementary material for: Feedback and Financial Incentives for Reducing Cell Phone Use While Driving: A Randomized Clinical Trial
Source: JAMA Netw Open. 2024 Jul 10;7(7):e2420218. doi: 10.1001/jamanetworkopen.2024.20218 (PMC11238027; doi:10.1001/jamanetworkopen.2024.20218)
Supplement: Supplement 2. — eFigure 1. CONSORT Flow Diagram eTable 1. Comparison of Eligible Customers Who Did and Did Not Enroll eFigure 2. Differences in Handheld Phone Use Relative to Control (Raw, Unadjusted Outcomes) eTable 2. Holm Sequential Analysis of 10 Pre-Specified Trial Group Comparisons (Raw, Unadjusted Outcomes) eFigure 3. Treatment Effects by Subgroup (Post-Intervention Period) [file jamanetwopen-e2420218-s002.pdf]

Supplementary Online Content

Delgado MK, Ebert JP, Xiong RA, et al. Feedback and financial incentives for reducing cell phone use while driving: a randomized clinical trial. *JAMA Netw Open*. 2024;7(7):e2420218. doi:10.1001/jamanetworkopen.2024.20218

eFigure 1. CONSORT Flow Diagram
eTable 1. Comparison of Eligible Customers Who Did and Did Not Enroll
eFigure 2. Differences in Handheld Phone Use Relative to Control (Raw, Unadjusted Outcomes)
eTable 2. Holm Sequential Analysis of 10 Pre-Specified Trial Group Comparisons (Raw, Unadjusted Outcomes)
eFigure 3. Treatment Effects by Subgroup (Post-Intervention Period)

This supplementary material has been provided by the authors to give readers additional information about their work.

© 2024 Delgado MK et al. *JAMA Network Open.*

eFigure 1. CONSORT flow diagram

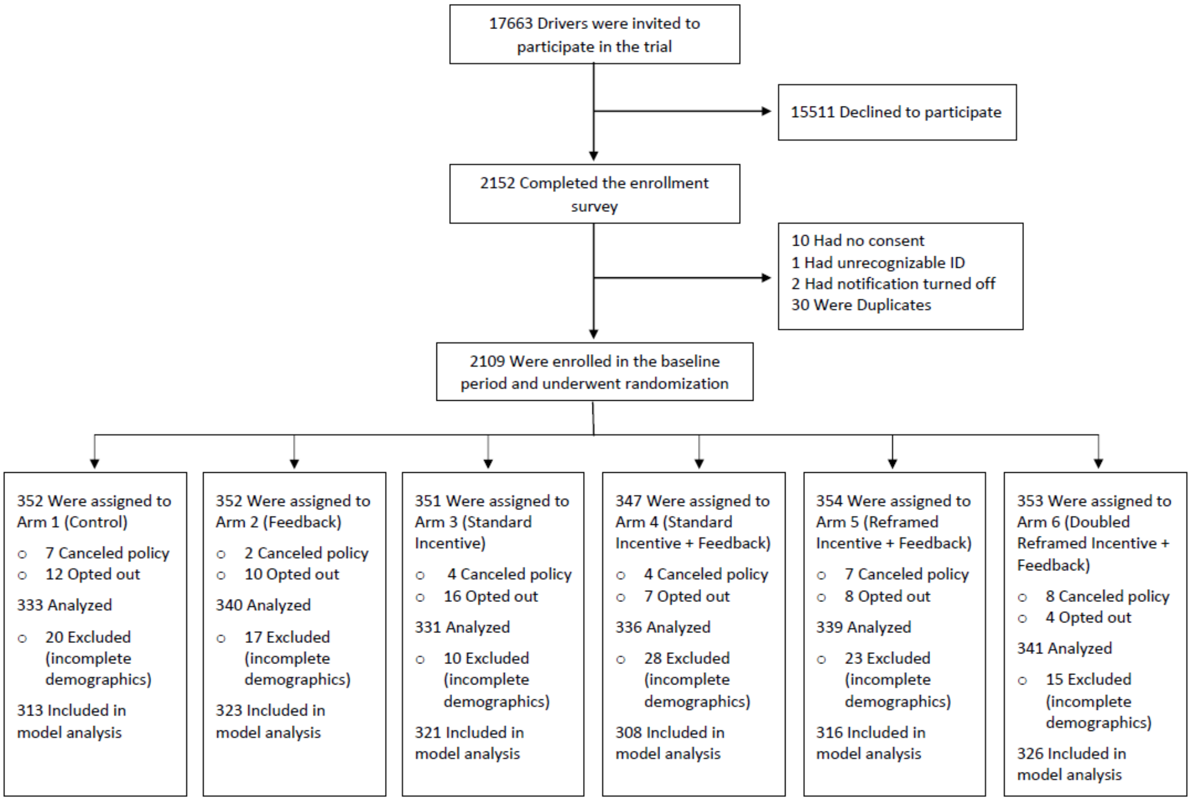

Participants randomly assigned to a study arm who did not cancel their policy or withdraw from the study were included in the intention-to-treat analyses (*n* = 2,020). The complete-case primary model reported in the article body excluded 113 participants who were missing covariate data; results for all 2,020 participants, unadjusted for covariates, are reported herein in the Supplementary Materials.

© 2024 Delgado MK et al. *JAMA Network Open.*

eTable 1. Comparison of eligible customers who did and did not enroll in study

		Non-Enrollees	Enrollees	P
n		15,511	2,109	
Age (mean (sd))		32.4 (11.23)	33.3 (11.05)	0.001
Age Group (%)	Under 20	1276 (8.2)	112 (5.3)	<.001
	20-24	2829 (18.2)	330 (15.6)	
	25-29	3752 (24.2)	518 (24.6)	
	30-34	2453 (15.8)	394 (18.7)	
	35-39	1729 (11.1)	248 (11.8)	
	40-44	1184 (7.6)	180 (8.5)	
	45-49	845 (5.4)	130 (6.2)	
	Over 50	1435 (9.3)	197 (9.3)	
Sex (%)	Female	8866 (57.2)	1431 (67.9)	<.001
	Male	6634 (42.8)	678 (32.1)	
Marital Status (%)	Married	2042 (13.2)	293 (13.9)	0.38
	Single	13461 (86.8)	1816 (86.1)	
Education (%)	No high school diploma or GED	337 (2.2)	33 (1.6)	<.001
	High school diploma or GED	3967 (25.6)	418 (19.8)	
	Vocational or Trade school degree	807 (5.2)	91 (4.3)	
	Currently in college	1435 (9.3)	170 (8.1)	
	Completed some college	2974 (19.2)	398 (18.9)	
	College degree	3284 (21.2)	529 (25.1)	
	Graduate work or graduate degree	1281 (8.3)	254 (12.0)	
Region (%)	Midwest	4031 (26.0)	564 (26.7)	0.11
	Northeast	2796 (18.0)	399 (18.9)	
	South	7414 (47.8)	1008 (47.8)	
	West	1249 (8.1)	136 (6.4)	
Driving Environment (%)	Rural	3329 (21.5)	419 (19.9)	0.24
	Suburban	9322 (60.1)	1295 (61.4)	
	Urban	2860 (18.4)	395 (18.7)	

Demographic characteristics of eligible Snapshot customers who did not vs. did enroll in the study. Data on non-enrollees were provided in aggregate by the company. Student's t-test was used to compare the two groups by mean age and chi-square tests were used to compare proportions for all other variables.

© 2024 Delgado MK et al. *JAMA Network Open.*

eFigure 2. Differences in handheld phone use relative to control (raw, unadjusted outcomes)

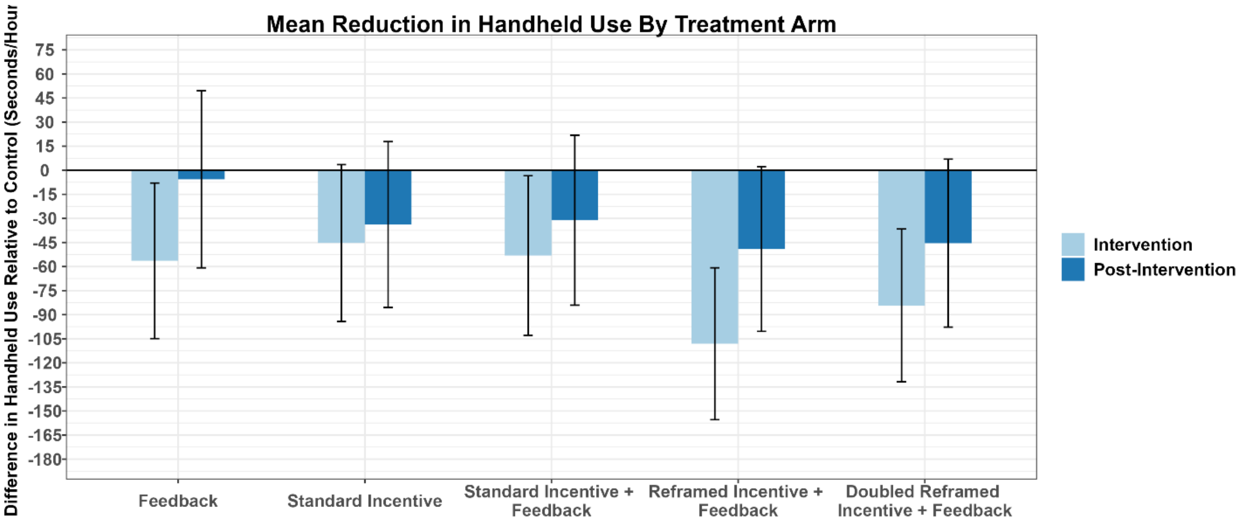

Plot of unadjusted differences in mean handheld use between each of the five intervention arms and control for the full sample (*n* = 2,020), with 95 percent confidence intervals, for both the intervention and post-intervention periods.

© 2024 Delgado MK et al. *JAMA Network Open.*

eTable 2. Holm sequential analysis of 10 pre-specified trial group comparisons (raw, unadjusted outcomes)

Comparison	Raw *P* value	Rank	Remaining contrasts	Holm threshold	Adjusted *P* value
2. Feedback vs. 1. Control	.023	3	8	.00625	.18
3. Standard Incentive vs. 1. Control	.069	6	5	.01	.35
4. Standard Incentive + Feedback vs. 1. Control	.036	5	6	.008	.22
5. Reframed Incentive + Feedback vs. 1. Control	.001	1	10	.005	<.001
6. Double Reframed Incentive + Feedback vs. 1. Control	<.001	2	9	.006	.005
3. Standard Incentive vs. 2. Feedback	.649	8	3	.017	>.99
4. Standard Incentive + Feedback vs. 3. Standard Incentive	.754	9	2	.025	>.99
4. Standard Incentive + Feedback vs. 2. Feedback	.894	10	1	.05	.89
5. Reframed Incentive + Feedback vs. 4. Standard Incentive + Feedback	.023	4	7	.007	.16
6. Double Reframed Incentive + Feedback vs. 5. Reframed Incentive + Feedback	.300	7	4	.013	>.99

Raw and adjusted *p*-values for the 10 intervention-period planned comparisons. Adjusted *p*-values may be directly compared to a two-sided alpha threshold of .05.

© 2024 Delgado MK et al. *JAMA Network Open.*

eFigure 3. Treatment effects by subgroup (post-intervention period)

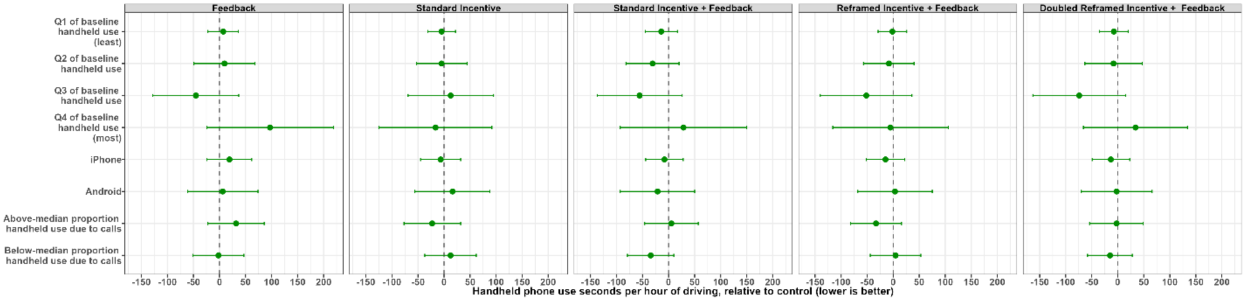

Mean adjusted differences in handheld phone use relative to control (seconds per hour of driving), with 95 percent confidence intervals, for the post-intervention period. Differences between treatment arms were estimated using a prespecified fractional logistic regression model adjusted for the prespecified covariates listed in Table 1. Details on the model are available in the statistical analysis plan included in the supplement.

© 2024 Delgado MK et al. *JAMA Network Open.*
