# Supplementary material for: Feedback and Financial Incentives for Reducing Cell Phone Use While Driving: A Randomized Clinical Trial
Source: JAMA Netw Open. 2024 Jul 10;7(7):e2420218. doi: 10.1001/jamanetworkopen.2024.20218 (PMC11238027; doi:10.1001/jamanetworkopen.2024.20218)
Supplement: Supplement 3. — Data Sharing Statement [file jamanetwopen-e2420218-s003.pdf]

Data Sharing Statement

Delgado. Feedback and Financial Incentives for Reducing Cell Phone Use While Driving. *JAMA Netw Open*. Published July 10, 2024.
doi:10.1001/jamanetworkopen.2024.20218

Data
Data available: Yes
Data types: Deidentified participant data, Data dictionary
How to access data: The analytic dataset and data dictionary will be made available in a public repository via a private link for approved users.
When available: With publication

Supporting Documents
Document types: Statistical/analytic code
How to access documents: The code used to analyze trial data is available at:
https://figshare.com/articles/dataset/_b_Feedback_and_financial_incentives_for_reducing_cellphone_use_while_driving_a_randomized_trial_b_/25992871
When available: With publication

Additional Information
Who can access the data: Data will be made available to researchers for research purposes.
Types of analyses: Data will be made available to researchers for research purposes.
Mechanisms of data availability: Requests for access to deidentifed data that support the findings can be made to the corresponding author at mucio.delgado@pennmedicine.upenn.edu. Restrictions apply to the availability of these data, which were used under license for this study. Deindentified data are available pending approval by the study authors with the permission of The Progressive Insurance Casualty Company
